# Supplementary material for: Generation of SIV-resistant T cells and macrophages from nonhuman primate induced pluripotent stem cells with edited CCR5 locus
Source: Stem Cell Reports. 2022 Mar 31;17(4):953–63. doi: 10.1016/j.stemcr.2022.03.003 (PMC9023799; doi:10.1016/j.stemcr.2022.03.003)
Supplement: Document S1. Figures S1–S4, Table S1, and Supplemental Experimental Procedures [file mmc1.pdf]

Stem Cell Reports, Volume *17*

## Supplemental Information

# Generation of SIV-resistant T cells and macrophages from nonhuman primate induced pluripotent stem cells with edited CCR5 locus

**Saritha S. D'Souza, Akhilesh Kumar, Jason Weinfurter, Mi Ae Park, John Maufort, Lihong Tao, HyunJun Kang, Samuel T. Dettle, Thaddeus Golos, James A. Thomson, Matthew R. Reynolds, and Igor Slukvin**

**Supplemental Materials for D'Souza et al.**

Generation of SIV resistant T cells and Macrophages from Nonhuman Primate Induced Pluripotent Stem Cells with Edited CCR5 locus

## Supplemental Experimental Procedures

### Reprogramming NHP cells and demonstration of pluripotency

Fibroblasts were obtained from skin punch biopsies of MCM CY1 with M3/M3 MHC genotype and MCM CY3 with M6/M6 MHC genotype, while peripheral blood samples were obtained from CY1, CY2, and CY3 with corresponding M3/M3, M1/M3 and M6/M6 MHC genotypes. Fibroblasts were obtained from skin biopsy and reprogrammed using a combination of oriP/EBNA-1 episomal vectors expressing the 6 reprogramming factors OCT4, KLF4, SOX2, MYC, NANOG and LIN28 (OKSMNL) by electroporation as described previously (D'Souza et al., 2016; Yu et al., 2009) to generate fibroblast iPSCs (Fib-iPSCs). Peripheral blood T cells were reprogrammed using the CytoTune™-iPS 2.0 Sendai Reprogramming Kit (ThermoFisher Scientific) to generate T-iPSCs (Seki et al., 2010). Briefly, peripheral blood mononuclear cells were first separated by Ficoll centrifugation and then MACS enriched for $CD3^+$ cells. These cells were then activated using CD3 and CD28 antibodies in the presence of 100U/ml IL-2 (Peprotech). For reprogramming, $0.5x10^6$ activated cells were resuspended in 0.3ml of 10% RPMI1640 medium (ThermoFisher) containing 4µg/ml of hexadimethrine bromide (MilliporeSigma) and 20µl each of the 3 Sendai viruses expressing KOS (KLF4–OCT3/4–SOX2), MYC, and KLF4, and incubated overnight at $37^oC$ in 5% $CO_2$. Cells were washed the following day and transferred onto mouse embryonic fibroblasts (MEFs). Colonies began to appear within 15 days and were subsequently transferred onto fresh MEFs for expansion and characterization. The iPSC lines (T-iPSCs and Fib-iPSCs) were maintained on MEFs in Primate ES cell medium (ReproCELL) supplemented with 4ng/ml bFGF (154 a.a.) (Peprotech) as previously described.(D'Souza *et al.*, 2016) Cells were passaged every 3-4 days using Collagenase Type IV (Life Technologies). Expression of pluripotency markers was analyzed by flow cytometry using SOX2 (Cell Signaling) and OCT3/4 (Santacruz Biotechnology) antibodies and the MACSQuant Analyzer 10 (Miltenyi) and FlowJo software (BD). Expression of pluripotency marker NANOG was analyzed by immunofluorescence using antibodies from Cell Signaling.

### RT-PCR analysis of CCR5 expression

RNA was isolated using the RNeasy mini Kit (Qiagen) from macrophages and T cells generated from wild type and CCR5-mut iPSCs. cDNA was transcribed using the QuantiTect reverse transcription kit (Qiagen) from all the samples and amplified by PCR using Q5 master mix (New England Biolabs) and forward-TGTGTCAATGGAACTCTTGAC and reverse-TCGTTTCGACACCGAAGCAG CCR5 primers.

**Supplemental References**

D'Souza, S.S., Maufort, J., Kumar, A., Zhang, J., Smuga-Otto, K., Thomson, J.A., and Slukvin, II (2016). GSK3beta Inhibition Promotes Efficient Myeloid and Lymphoid Hematopoiesis from Non-human Primate-Induced Pluripotent Stem Cells. Stem Cell Reports *6*, 243-256. 10.1016/j.stemcr.2015.12.010.

Seki, T., Yuasa, S., Oda, M., Egashira, T., Yae, K., Kusumoto, D., Nakata, H., Tohyama, S., Hashimoto, H., Kodaira, M., et al. (2010). Generation of induced pluripotent stem cells from human terminally differentiated circulating T cells. Cell Stem Cell *7*, 11-14. 10.1016/j.stem.2010.06.003.

Yu, J., Hu, K., Smuga-Otto, K., Tian, S., Stewart, R., Slukvin, II, and Thomson, J.A. (2009). Human induced pluripotent stem cells free of vector and transgene sequences. Science *324*, 797-801. 10.1126/science.1172482.

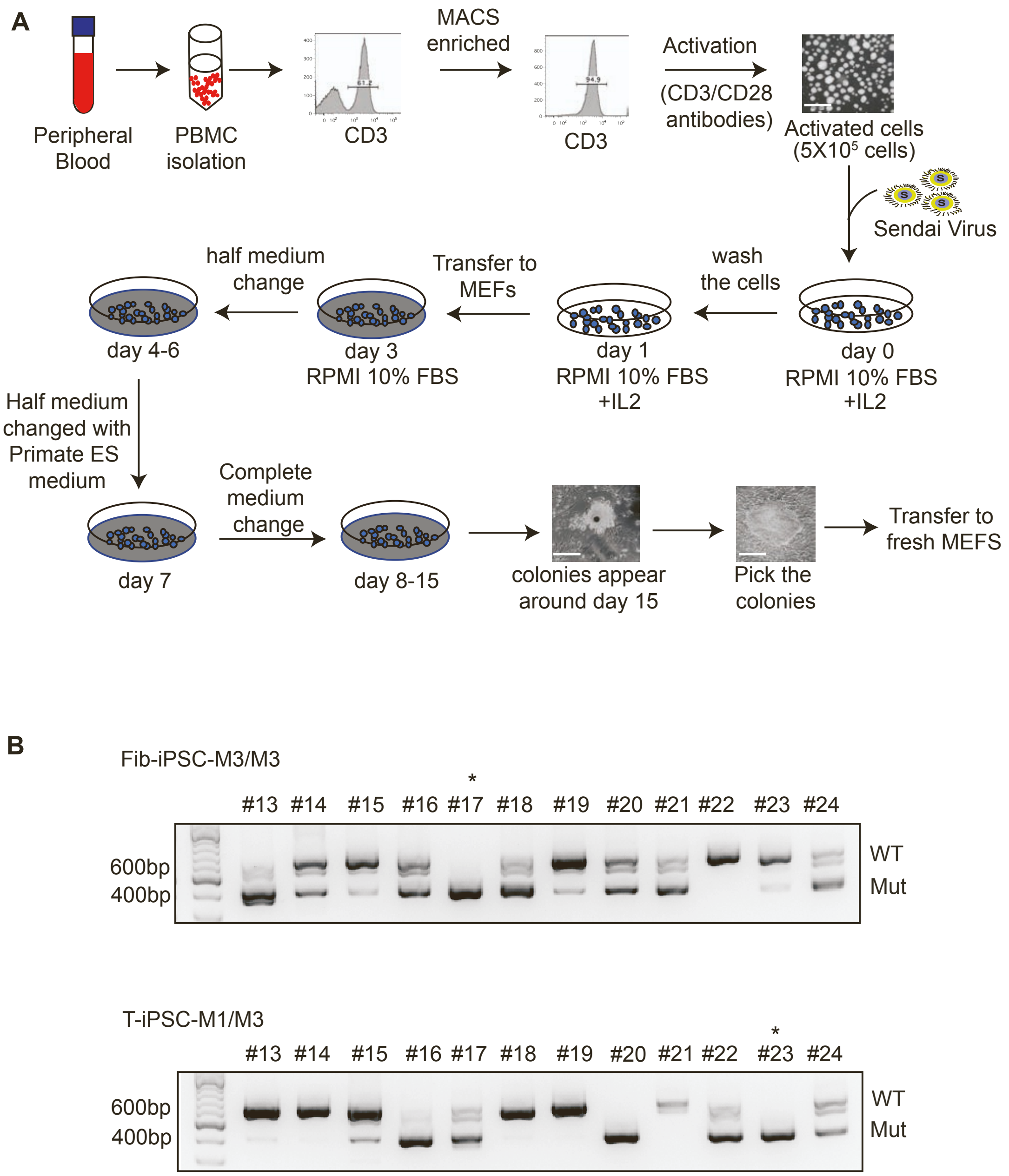


**Supplementary Figure S1, related to Figure 1.** Generation of CCR5mut iPSCs from MCM fibroblasts and T cells. (A) Schematic diagram of generation of T-iPSCs from peripheral blood T cells using Sendai virus kit. Scale bar in activated T cell images is 300 μm. Scale bar for colonies at day 15 is 150 μm and for pick up colonies is 740 μm. (B) Genomic PCR to detect deletion within CCR5 locus. * denotes clones selected in these studies.

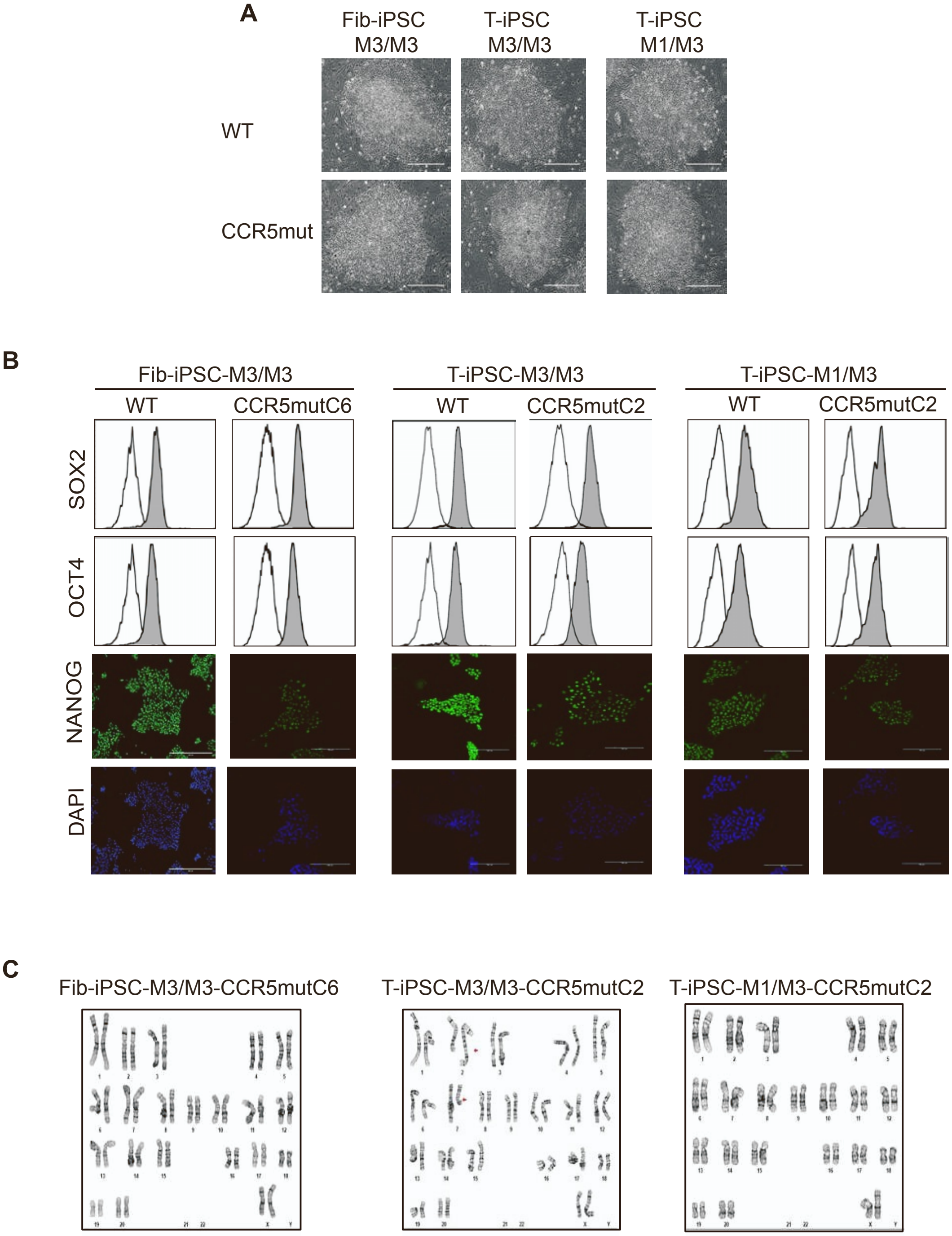


**Supplementary Figure S2, related to Figure 1.** Generation and characterization of MCM-iPSCs. (A) Phase contrast images of WT and CCR5mut colonies on MEFs. Scale bar is 200 µm. (B) Expression of pluripotency markers by flow cytometry (OCT4 and SOX2) and by immunofluorescence (NANOG) in generated wild type and CCR5mut iPSC lines. Scale bars for WT Fib-iPSC-M3/M3 images are 400 µm. Scale bars for all other images are 100 µm. (C) Karyotype of CCR5mut iPSCs.

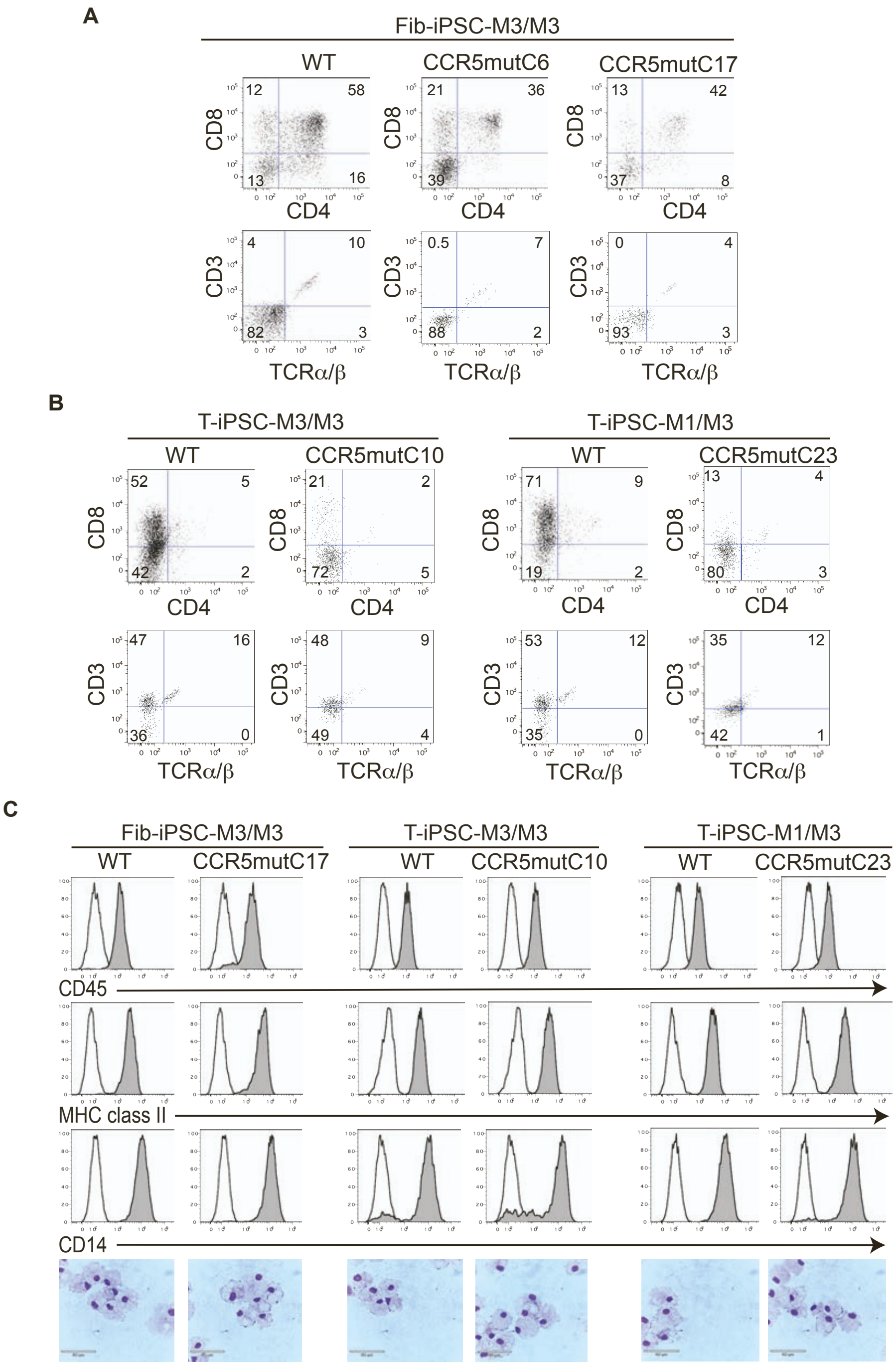


**Supplemental Figure S3, related to Figure 2 and 3.** Generation of T cells and macrophages from CCR5mut clones. (A) CCR5mutC6 and C17 clones from fib-iPSCs and (B) C10 and C23 CCR5mut clones from T-iPSC-M3/M3 and M1/M3 were differentiated into T cells. Expression of T cell markers was shown on $CD45^+$ gated cells after 3 weeks of culture of iPSC-derived MHP on OP9-DLL4 in the presence of SCF, IL7 and FLT3L. (C) Day 10 $CD34^+CD45^+$ MHPs from mutant and wild type NHP iPSCs were differentiated to macrophages. The phenotype and morphology of the cells was confirmed by flow cytometry and Wright stain of cytospins. Scale bar is 50 µm.

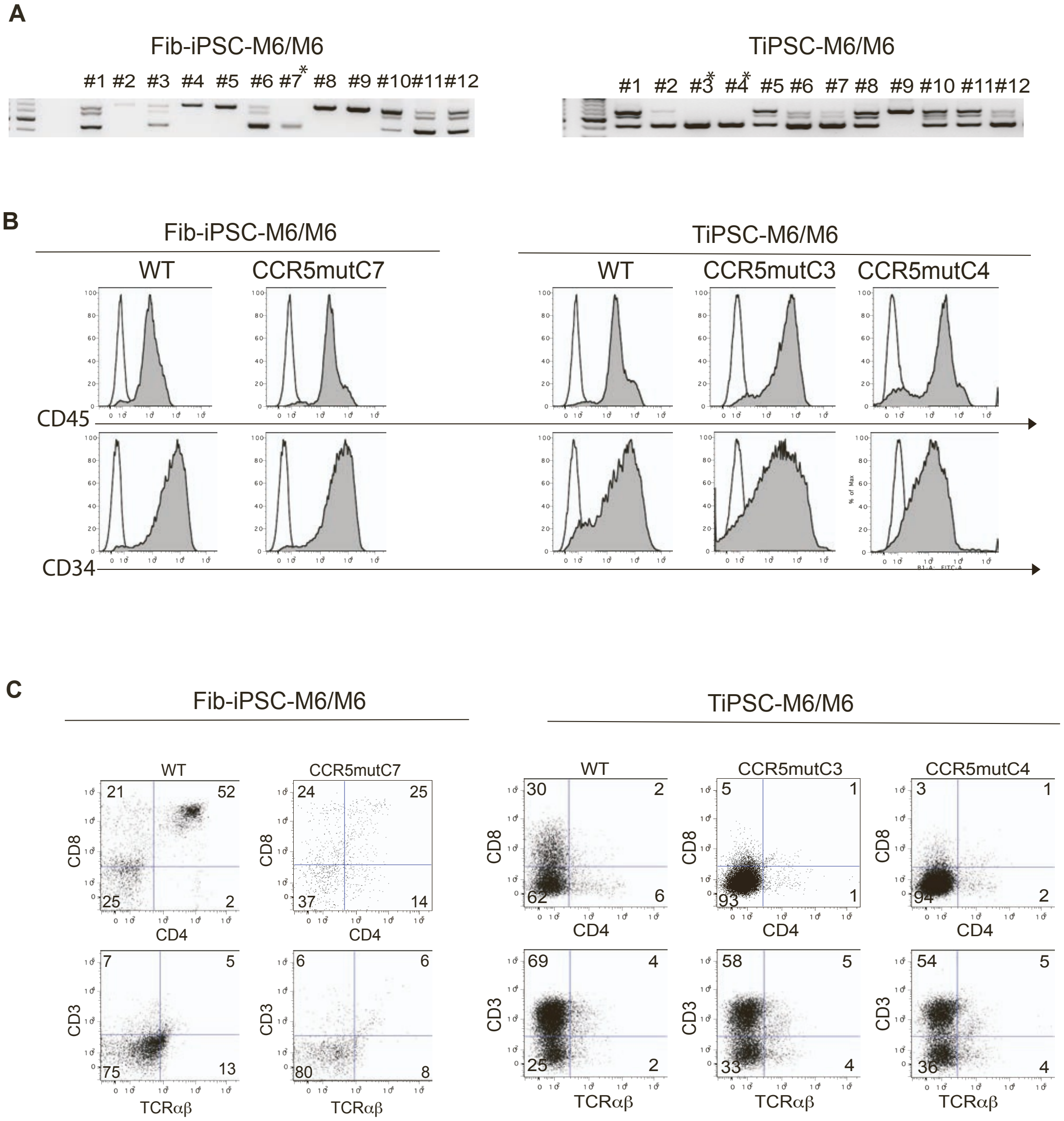


**Supplemental Figure S4, related to Figure 1, 2 and 3.** Generation of fib- and T-iPSCs from MCM with M6/M6 genotype and their hematopoietic differentiation. (A) Genomic DNA was isolated from single cell clones obtained after CCR5 editing and analyzed by PCR to detect deletion within the CCR5 locus. * Denotes the clones selected for further analysis. (B) Both wild type and CCR5mut, fib- and T-iPSCs efficiently generate $CD34^{+}CD45^{+}$ hematopoietic progenitors as analyzed by flow cytometry of floating cells collected on day10 of iPSC/OP9 cocultures. (D) $CD34^{+}CD45^{+}$ cells from WT and CCR5mut iPSCs were cultured on OP9-DLL4 in the presence of SCF, IL7 and FLT3L for 3 weeks and floating cells were analyzed by flow cytometry after gating on $CD45^{+}$ cells. Control staining with appropriate isotype matched antibodies were included to establish a threshold for positive staining. The graphs are representative of at least 3 independent experiments.

**Supplementary Table S1. List of antibodies used in this study**

| Name | Clone | Vendor | Cat. Number |
|---|---|---|---|
| Anti NHP CD45 | MB4-6D6 | Miltenyi Biotech | 130-119-764 |
| Anti human CD34 | 563 | BD Biosciences | 550619 |
| Anti human CD3ε | SP34 | BD Biosciences | 556612 |
| Anti human CD4 | L200 | BD Biosciences | 550630 |
| Anti human CD5 | UCHT2 | Biolegend | 300611 |
| Anti human CD7 | MT701 | BD Biosciences | 340656 |
| Anti rat TCRα/β | R73 | Biolegend | 201110 |
| Anti human CD8 | SK1 | Biolegend | 980904 |
| Anti human CD14 | M5E2 | BD Biosciences | 555399 |
| Anti human MHC class II | L243 | BD Biosciences | 340689 |
| Anti monkey CD3-1 | monoclonal | Mabtech | 3610-1-50 |
| Anti human CD28-A | monoclonal | Mabtech | 3608-1-50 |
| Oct 3/4 | monoclonal | Santa Cruz | Sc-5279 |
| Donkey anti mouse IgG (H+L) Alexa Fluor®488 | polyclonal | Invitrogen | A-31570 |
| Nanog | monoclonal | Cell Signaling Technology | 4903 |
| Donkey anti rabbit IgG (H+L) Alexa Fluor®488 | polyclonal | Invitrogen | A-21206 |
| Sox 2 | monoclonal | Cell Signaling Technology | 3579 |
| Donkey anti rabbit IgG (H+L) Alexa Fluor®555 | polyclonal | Invitrogen | A-31572 |
