# Supplementary material for: Generation of SIV-resistant T cells and macrophages from nonhuman primate induced pluripotent stem cells with edited CCR5 locus
Source: Stem Cell Reports. 2022 Mar 31;17(4):953–63. doi: 10.1016/j.stemcr.2022.03.003 (PMC9023799; doi:10.1016/j.stemcr.2022.03.003)
Supplement: Document S2. Article plus Supplemental information [file mmc2.pdf]

Stem Cell Reports

ISSCR

Resource

OPEN ACCESS

Generation of SIV-resistant T cells and macrophages from nonhuman primate induced pluripotent stem cells with edited CCR5 locus

Saritha S. D'Souza,[1,9] Akhilesh Kumar,[1,9] Jason Weinfurter,[2,9] Mi Ae Park,[1] John Maufort,[3] Lihong Tao,[1] HyunJun Kang,[1] Samuel T. Dettle,[1] Thaddeus Golos,[1,4,5] James A. Thomson,[3,6] Matthew R. Reynolds,[1,2,10] and Igor Slukvin[1,7,8,10,*]

[1]Wisconsin National Primate Research Center, University of Wisconsin-Madison, Madison, WI 53715, USA
[2]Department of Pathobiological Sciences, School of Veterinary Medicine, University of Wisconsin, Madison, WI 53706, USA
[3]Morgridge Institute for Research, Madison, USA
[4]Department of Comparative Biosciences, University of Wisconsin-Madison, Madison, WI 53706 USA
[5]Department of Obstetrics and Gynecology, University of Wisconsin-Madison, Madison, WI 53705 USA
[6]Department of Molecular, Cellular, and Developmental Biology, University of California, Santa Barbara, CA 93106, USA
[7]Department of Cell and Regenerative Biology, University of Wisconsin-Madison, Madison, WI 53706, USA
[8]Department of Pathology and Laboratory Medicine, University of Wisconsin-Madison, Madison, WI 53792, USA
[9]These authors contributed equally
[10]Senior authors
*Correspondence: islukvin@wisc.edu
https://doi.org/10.1016/j.stemcr.2022.03.003

SUMMARY

Adoptive therapies with genetically modified somatic T cells rendered HIV resistance have shown promise for AIDS therapy. A renewable source of HIV-resistant human T cells from induced pluripotent stem cells (iPSCs) would further facilitate and broaden the applicability of these therapies. Here, we report successful targeting of the *CCR5* locus in iPSCs generated from T cells (T-iPSCs) or fibroblasts (fib-iPSCs) from Mauritian cynomolgus macaques (MCM), using CRISPR-Cas9 technology. We found that *CCR5* editing does not affect hematopoietic and T cell differentiation potentials of fib-iPSCs. However, T-iPSCs with edited CCR5 lost their capacity to differentiate into $CD4^+CD8^+$ T cells while maintaining myeloid differentiation potential. T cells and macrophages produced from *CCR5*-edited MCM iPSCs did not support replication of the CCR5-tropic simian immunodeficiency viruses SIVmac239 (T cell tropic) and SIVmac316 (macrophage-tropic). Overall, these studies provide a platform for further exploration of AIDS therapies based on gene-edited iPSCs in a nonhuman primate model.

INTRODUCTION

Adoptive T cell therapies with *in vitro* expanded genetically modified T cells have been considered a valuable strategy to treat and cure HIV (Hale et al., 2017; Lam and Bollard, 2013; Patel et al., 2018; Sung et al., 2018). However, T cell exhaustion along with complicated logistics for generation and delivery of genetically modified T cells hampers the broader application of these technologies. Genetic modification of induced pluripotent stem cells (iPSCs) to introduce HIV-resistance and/or anti-HIV molecules, can serve as a versatile and scalable source for off-the-shelf adoptive T cell therapies. In addition, reprogramming of HIV-specific cytotoxic T lymphocytes (CTLs) from HIV-infected patients allows for capturing the specific T cell receptors (TCRs) within the iPSC genome and generating "rejuvenated" antigen-specific CTLs from these iPSCs (Ando and Nakauchi, 2017; Nishimura et al., 2013).

To enable evaluation of iPSC-based technologies in a preclinical HIV infection model, we explored the feasibility of interrupting the *CCR5* locus in iPSCs from nonhuman primate (NHP) sources and *de novo* generating simian immunodeficiency (SIV)-resistant T cells and macrophages from these modified iPSCs. In these studies, we used Mauritian cynomolgus macaques (MCMs) that have a limited major histocompatibility complex (MHC) diversity (Budde et al., 2010; Wiseman et al., 2007, 2013), and could be used to assess adoptive cellular therapies, including T cell therapies in an MHC-defined setting (Greene et al., 2013). iPSCs were generated from fibroblasts and peripheral blood T cells. To successfully disrupt *CCR5*, we designed two CCR5 synthetic guide RNAs (gRNAs) to target sequences within exon 2, including a 24-base pair (bp) deletion region that was previously found to prevent functional CCR5 expression in NHPs (Chen et al., 1998). Using this approach, we generated SIV-resistant T cells and macrophages from NHP iPSCs, thus laying a foundation for further exploration of iPSC technology for AIDS treatment in an NHP preclinical model. In addition, we noted an impaired capacity of iPSCs generated from T cells (T-iPSCs) to redifferentiate into T cells, especially following biallelic *CCR5* disruption. This finding should be taken into consideration when designing strategies for HIV immunotherapies using rejuvenated T cells.

This is an open access article under the CC BY license (http://creativecommons.org/licenses/by/4.0/).

RESULTS

Editing of *CCR5* locus in iPSCs from MCM fibroblasts and T cells

Using oriP/EBNA-1 episomal plasmids expressing the six reprogramming factors OCT4, KLF4, SOX2, MYC, NANOG, and LIN28 (OKSMNL), we successfully established an iPSC line from fibroblasts of MHC homozygous MCMs with M3/M3 (fib-iPSC-M3/M3). T-iPSC lines from MCMs with an MHC M3/M3 (T-iPSC-M3/M3) and an MHC M1/M3 (T-iPSC-M1/M3) genotype were generated from peripheral blood T cells using the CytoTune-iPS 2.0 Sendai Reprogramming Kit (Figures 1A and S1A). Generated fib-iPSCs and T-iPSCs exhibited typical NHP embryonic stem cell (ESC) morphology and expressed pluripotency markers OCT4, SOX2, and NANOG (Figures S2A and S2B).

To disrupt *CCR5*, we used two gRNAs to target sequences within exon 2, including a 24-bp deletion region known to be essential for expressing functional CCR5 in NHPs (Chen et al., 1998) (Figure 1B). We have shown in prior studies with human iPSCs that dual single guide RNAs (sgRNAs) were more efficient in introducing *CCR5* gene editing in human iPSCs as compared with a single sgRNA (Kang et al., 2015). By genomic PCR, we found that the dual gRNAs resulted in 66% of CCR5 mutations (CCR5mut) in fib-iPSC-M3/M3, 50% in T-iPSC-M3/M3, and 83% in T-iPSC M1/M3. Of these, 19% of the clones demonstrated biallelic mutation in the fib-iPSC-M3/M3 and 33% in T-iPSC M3/M3 and 15% in T-iPSC M1/M3 (Figures 1C and S1B). Following *CCR5* editing, we established CCR5mut iPSC lines from each wild-type (WT) iPSC:CCR5-mutC6 from fib-iPSC-M3/M3, CCR5mutC2 from T-iPSC-M3/M3, and CCR5mutC2 from T-iPSC-M1/M3 lines. CCR5mut iPSCs retained pluripotent morphology and expression of pluripotency markers OCT4, SOX2, and NANOG (Figures S2A and S2B). Karyotyping revealed a normal karyotype for CCR5mutC6-fib-iPSC-M3/M3 and CCR5mutC2-T-iPSC-M1/M3. However, CCR5mutC2-T-iPSC-M3/M3 demonstrated a balanced translocation between the long (q) arms of chromosomes 2 and 7 (Figure S2C). This translocation was detected in several *CCR5* mutated clones, suggesting that it was introduced during reprogramming, rather than during *CCR5* editing.

Generation of T cells and macrophages from CCR5mut iPSCs

To induce hematopoietic differentiation, we used the OP9 co-culture system with CHIR99021 and vascular endothelial growth factor (VEGF) to efficiently induce mesoderm and definitive hematopoiesis (D'Souza et al., 2016) (Figure 2A). Floating cells collected from day 10 of iPSCs/OP9 co-culture were analyzed by flow cytometry. All iPSCs, including WT and CCR5mut fib- and T-iPSCs efficiently produced multipotent hematopoietic progenitors (MHPs) with more than 90% of floating cells expressing CD34 and CD45 (Figure 2B).

To induce T cell differentiation, we collected day 10 $CD34^{+}CD45^{+}$ MHPs and cultured them on OP9-DLL4 in the presence of interleukin (IL)-7, FLT3 ligand, and stem cell factor (SCF) according to our protocol, which generates functional T cells with rearranged TCR (D'Souza et al., 2016; Kumar et al., 2019). In these cultures, MHPs gave rise to $CD5^{+}CD7^{+}$ lymphoid progenitors and eventually to $CD4^{+}CD8^{+}$ T cells (Figure 2C). As reported in prior studies with human T-iPSCs (Nishimura et al., 2013), MCM T-iPSCs show surface CD3 expression very early during differentiation. As shown in Figure 2C, surface CD3 expression was already detected in T-iPSC cultures at 2 weeks of T cell differentiation. However, T cells from fib-iPSCs demonstrated mostly intracellular CD3 expression with negligible surface CD3 expression at this stage of differentiation (Figure 2C). No differences in T cell differentiation were observed between WT fib-iPSC-M3/M3 and CCR5mutC6-fib-iPSC-M3/M3. In contrast, $CD4^{+}CD8^{+}$ T cell differentiation of MHPs from T-iPSCs was less efficient as compared with fib-iPSCs and both CCR5mutC2-T-iPSC-M1/M3 and CCR5mutC2-T-iPSC-M3/M3 iPSCs failed to produce $CD4^{+}CD8^{+}$ T cells (Figure 2C). To ensure that these differences were not clone- and individual-dependent, we established three additional CCR5mut iPSCs from the WT fib- and T-iPSC lines described above: CCR5mutC17-fib-iPSC-M3/M3, CCR5mutC10-T-iPSC-M3/M3-, and CCR5mutC23-T-iPSC-M1/M3; and generated five additional WT and CCR5mut fibroblast and T-iPSCs from monkeys with the MHC M6/M6 genotype: fib-iPSC-M6/M6, T-iPSC-M6/M6, CCR5mutC7-fib-iPSC-M6/M6, CCR5mutC3-T-iPSC-M6/M6, and CCR5mutC4-T-iPSC-M6/M6. As shown in Figures S3 and S4, all these lines demonstrated a similar pattern of T cell differentiation, thus confirming that CCR5 knockout in T-iPSCs impairs their differentiation into $CD4^{+}CD8^{+}$ T cells.

For macrophage differentiation, the day 10 MHPs were collected and plated on ultralow attachment plates in Iscove's modified Dulbecco's medium (IMDM) with 10% fetal bovine serum (FBS), macrophage colony-stimulating factor (M-CSF), and IL-1β (Figure 3A). Cells collected after 5 to 7 days displayed typical macrophage morphology and phenotype (Figure 3B). We did not observe significant differences in the macrophage differentiation between WT and CCR5mut-fib-iPSCs and CCR5mut-T-iPSCs from multiple CCR5mut iPSC lines (Figures 3B and S3B). Genomic PCR analysis confirmed that biallelic CCR5 mutations were maintained in both the fib-iPSC and T-iPSC derivatives following hematopoietic differentiation, including multipotent $CD34^{+}CD45^{+}$ MHPs, macrophages, and T cells (Figures 3C and 3D). In addition, the presence of native CCR5

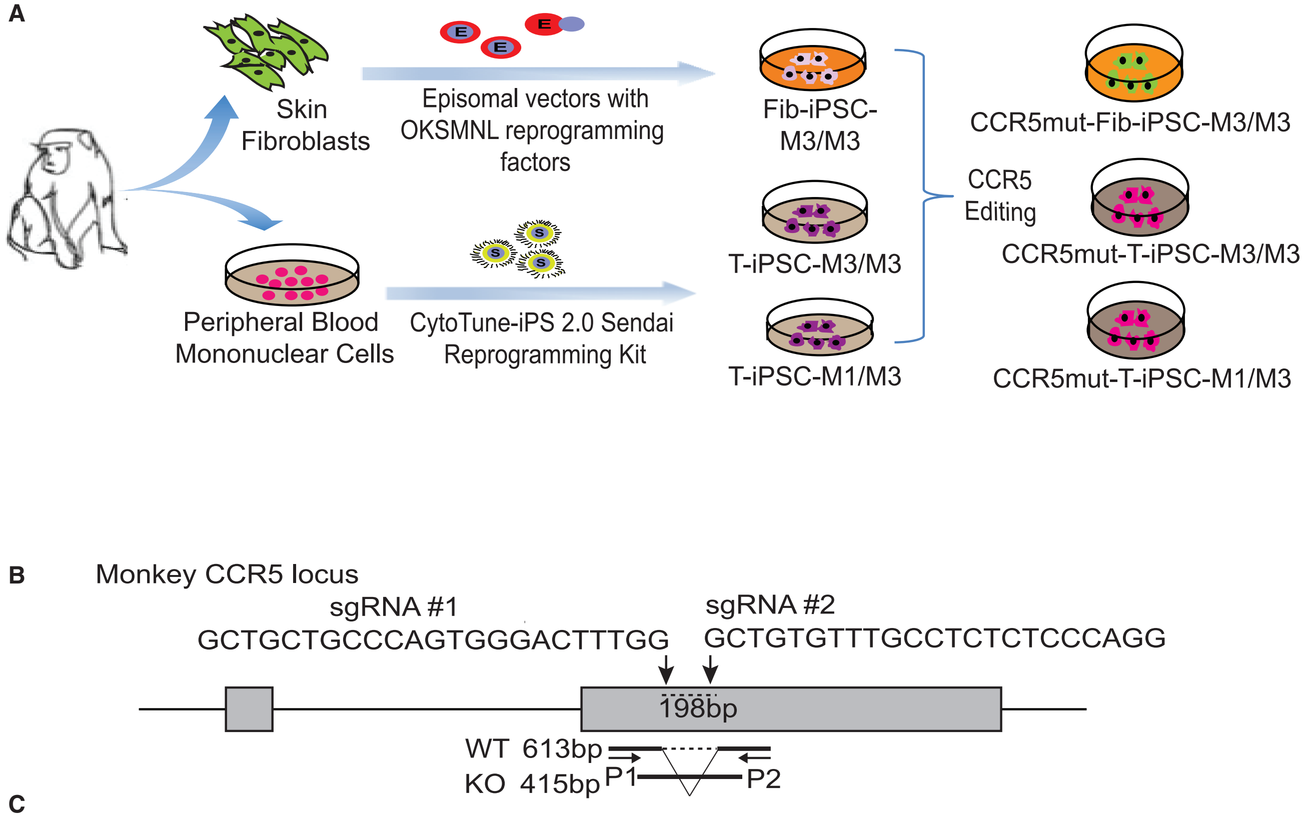

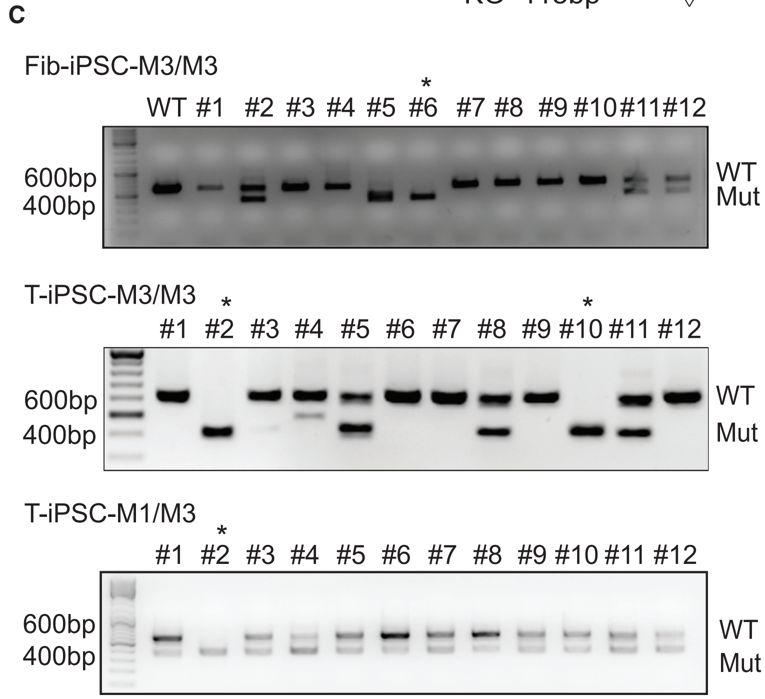

Cell Type	Total clones picked	Total mutant clones		Biallelic clones among mutant clones	
		actual no.	%	actual no.	%
Fib-iPSC-M3/M3	24	16	66	3	19
T-iPSCs-M3/M3	12	6	50	2	33
T-iPSCs-M1/M3	24	20	83	3	15

Figure 1. Generation of CCR5mut iPSCs from MCM fibroblasts and T cells
(A) Schematic diagram of the experimental design.
(B) Schematic representation showing the target site, sequences of the two gRNAs used to delete the 198-bp *CCR5* fragment, and position of PCR primers (P1 and P2) used to detect deletion.
(C) Genomic PCR to detect deletion within *CCR5* locus. *Denotes clones selected in these studies.
See also Figures S1, S2, and S4.

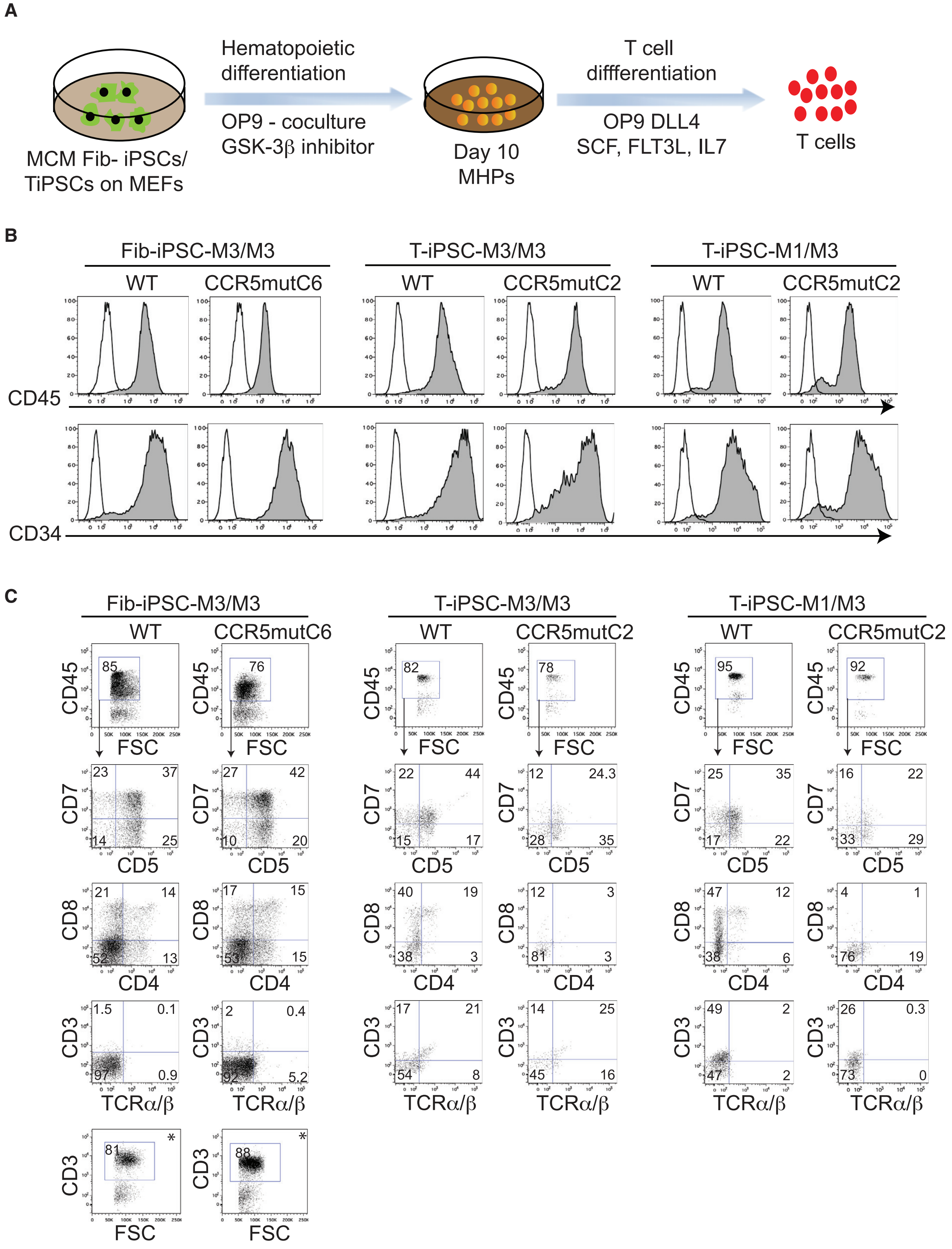

(legend on next page)

mRNA in macrophages and T cells from WT iPSCs and the lack of CCR5 mRNA expression in these cells from CCR5mut iPSCs was confirmed by RT-PCR (Figure 3E).

Resistance of CCR5mut macrophages and T cells to SIV infection

The aim of our study was to generate *CCR5* gene-disrupted NHP iPSCs that when differentiated to T cells and macrophages are resistant to CCR5-tropic SIV infection. Since CCR5mut-T-iPSCs failed to differentiate into $CD4^{+}CD8^{+}$ T cells, we only used fib-iPSC-M3/M3 for these studies. To evaluate protection from infection, we challenged T cells and macrophages from WT and CCR5mut-fib-iPSCs with the T cell-tropic SIVmac239 and macrophage-tropic SIVmac316 open SpX (1) virus isolates. As shown in Figure 4, SIV replication, as judged by SIV Gag p27 production, was observed at 4 days in T cells and macrophage cultures from WT fib-iPSCs. In contrast, CCR5mut T cell and macrophage cultures were resistant to productive SIV infection (Figure 4). The productive SIVmac239 infections in our WT fib-iPSC-derived $CD4^{+}CD8^{+}$ cultures were lower than productive infections reported in peripheral blood T cells (Gautam et al., 2007; Naidu et al., 1988; Sacha and Watkins, 2010). These findings are consistent with a previous report of thymocytes supporting low levels of CCR5 tropic HIV replications (Pedroza-Martins et al., 1998). Nevertheless, we observed distinct differences in the susceptibility of WT and CCR5mut iPSC-derived $CD4^{+}CD8^{+}$ T cells to SIVmac239 infections. Although we detected ~100 pg of p27/mL of supernatant 1-day post-magnetofection in the WT fib-iPSC-derived T cell cultures, we believe this reflects residual virus bound to cell surfaces rather than significant levels of *de novo* SIVmac239 production. Overall, these results demonstrate that *CCR5* gene-disrupted T cells and macrophages successfully generated from *CCR5* gene altered MCM iPSCs were protected from CCR5-tropic SIV challenge.

DISCUSSION

iPSCs derived from somatic cells offer an attractive strategy for the generation of HIV-resistant immune cells for adoptive immunotherapies. In this study, we demonstrated the feasibility of reprogramming T cells from NHPs to pluripotency and generation of SIV-resistant *CCR5*-mutated T cells and macrophages from fib-iPSCs obtained from MCMs. MCMs are descended from a small founder population and have a very limited MHC diversity consisting of only seven common haplotypes M1-M7 (Budde et al., 2010; Wiseman et al., 2007), therefore making it possible to rapidly select MHC identical, MHC homozygous, and MHC heterozygous animals to explore the utility of MHC homozygous iPSC banking for allogeneic immunotherapies using cells with beneficial MHC match in a preclinical NHP model.

Gene editing to inactivate the *CCR5* gene using nucleases has shown promising results toward developing a functional HIV cure (Mock et al., 2015; Mussolino et al., 2014; Tebas et al., 2014; Xu et al., 2019). Three main classes of nucleases, including ZFNs, TALENs, and CRISPR-Cas9 are routinely used to precisely delete or insert specific DNA sequences into the genome. Of these nucleases, the CRISPR-Cas9 system has demonstrated higher cleavage efficiency compared with ZFNs and TALENs (Chen et al., 2013; Fu et al., 2014; Tycko et al., 2016; Zhang et al., 2016). In this study, we employed dual gRNA-guided Cas9 systems to specifically target the *CCR5* region affected by Δ24 mutation, which prevents R5 lentiviral infections in macaques (Chen et al., 1998) and is functionally equivalent to the *CCR5Δ32* mutation in humans (Liu et al., 1996; Samson et al., 1996). Using the dual gRNA CRISPR/Cas9 system, we achieved up to a 33% biallelic mutation in NHP iPSCs. Our results are consistent with several other reports showing improved editing efficiency using dual gRNA in primary human $CD4^{+}$ T cells, $CD34^{+}$ hematopoietic progenitor cells, and iPSCs (Kang et al., 2015; Mandal et al., 2014; Xiao et al., 2019). T cells and macrophages produced from CCR5mut-fib-iPSCs were resistant to SIV challenge, implying that immune cells derived from the NHP iPSCs are useful for continued studies of novel immunotherapies for HIV in a preclinical NHP model. In addition, gene-editing technologies can be used to modify other genes in iPSCs that are essential for HIV/SIV replication, even producing NHP cells that support HIV replication. For

Figure 2. Generation of T cells from CCR5mut MCM iPSCs

(A) Schematic representation of hematopoietic differentiation of fib- and T-iPSCs to MHPs and their further differentiation to T cells.

(B) Both WT and CCR5mut iPSCs efficiently differentiated into MHPs as analyzed by flow cytometry of floating cells collected from iPSC/OP9 co-cultures on day 10 of differentiation.

(C) Day10 MHPs from WT and CCR5mut iPSCs were cultured on OP9-DLL4 in the presence of SCF, IL-7, and FLT3L for 2 weeks, and the floating cells were analyzed by flow cytometry after gating $CD45^{+}$ cells. *Intracellular CD3 was analyzed in WT and CCR5mut fib-iPSCs differentiation cultures.

In (B) and (C), control staining with the appropriate isotype matched mouse monoclonal antibody were included to establish a threshold for positive staining. The graphs are representative of at least three independent experiments.

See also Figures S3 and S4.

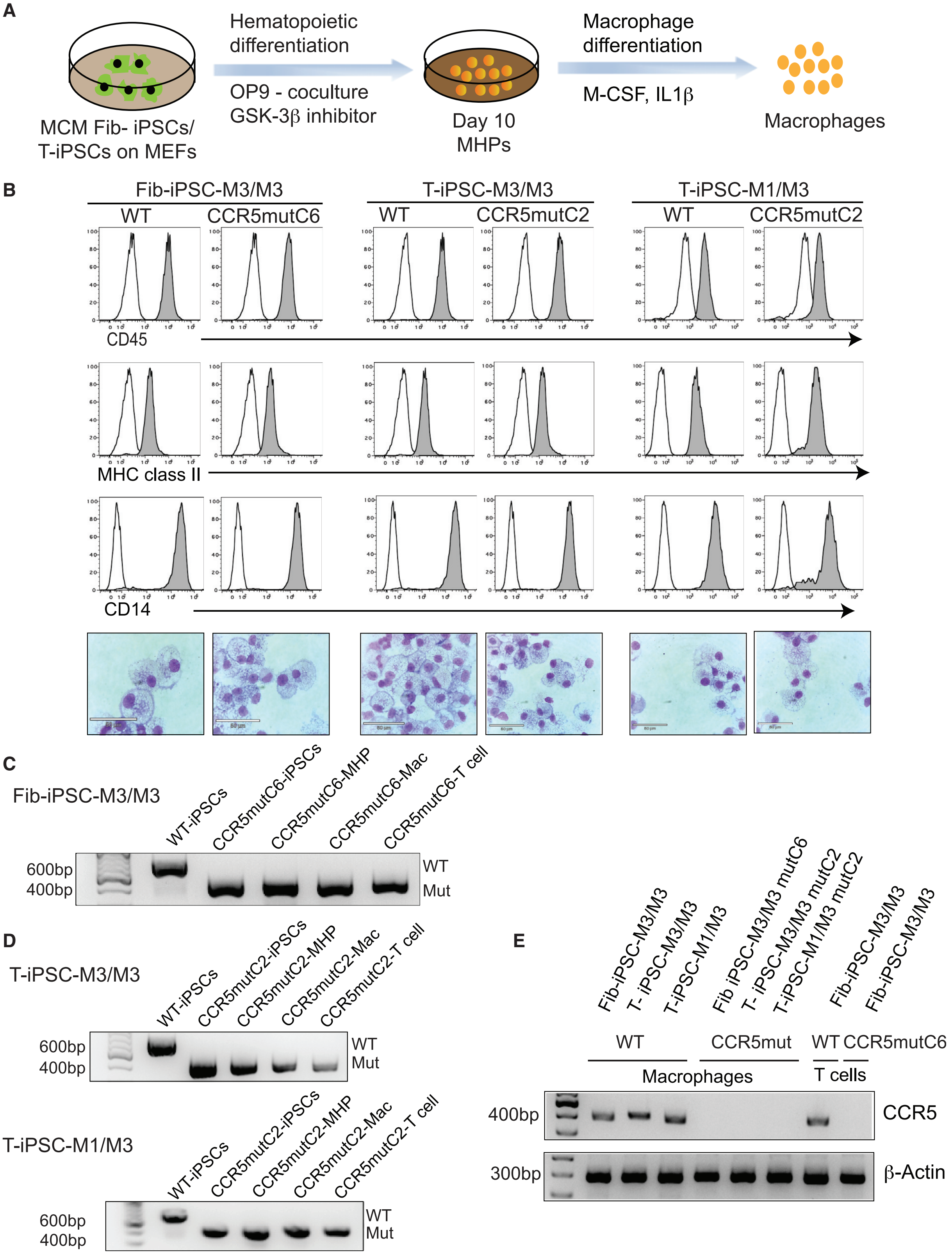

(legend on next page)

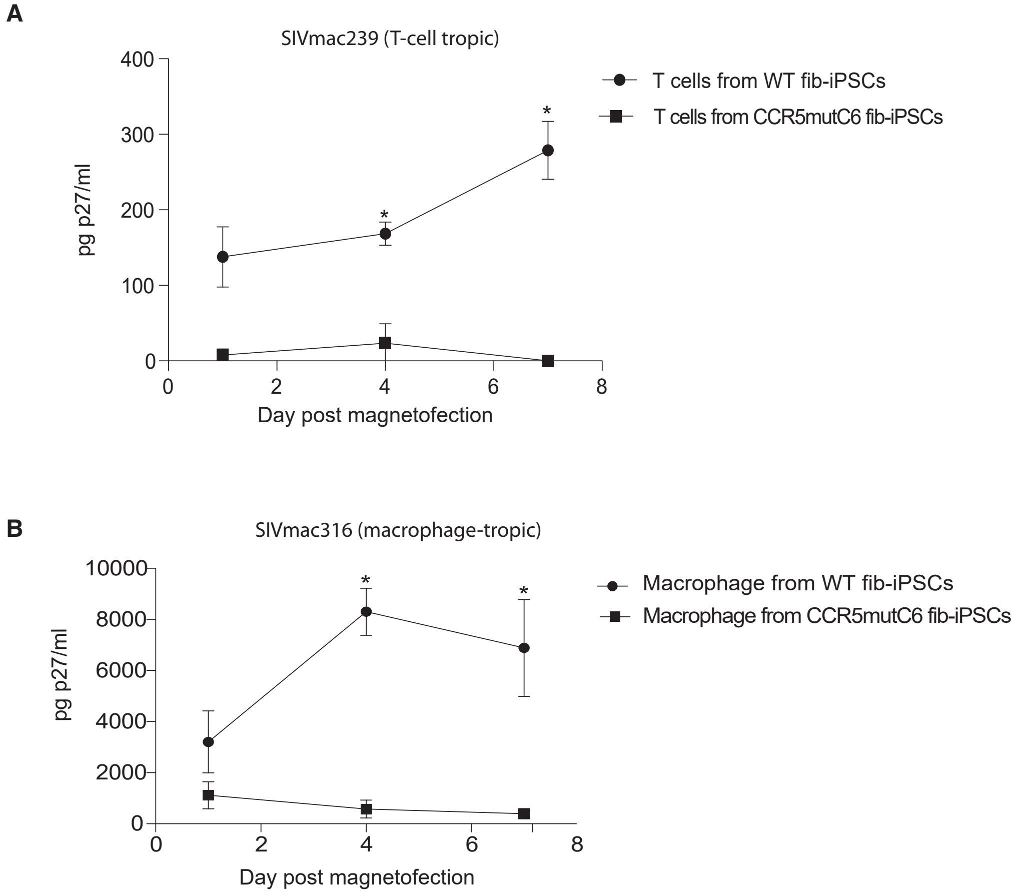

Figure 4. CCR5mut T cells and macrophages resist SIV infection

(A and B) Fib-iPSC-derived mutant and WT T cells (A) and macrophages (B) were incubated with the T-cell-tropic SIVmac239 and macrophage-tropic SIVmac316, respectively. Virus production was measured by collecting cell culture supernatant on days 1, 4, and 7 post-infection and performing SIV Gag p27 ELISAs. The graph is representative of three independent experiments performed in triplicate. The error bars represent the SE of each time point. *Denotes $p < 0.05$.

example, a recent study knocked out *TRIM5* from NHP iPSCs (Iwamoto et al., 2021), a gene encoding a restriction factor that blocks cross-species retrovirus infections, yielding NHP macrophages that are permissive to HIV infection, further demonstrating iPSC technologies' utility in modeling HIV infections.

Generating T cells from T-iPSCs opens opportunities for T cell rejuvenation and unlimited manufacturing of T cells with an antigen-specific monoclonal TCR (Iriguchi et al., 2021; Karagiannis et al., 2016; Nishimura et al., 2013; Trotman-Grant et al., 2021; Vizcardo et al., 2013). The feasibility of this approach for generating therapeutic cells has been demonstrated in murine and human studies, where rejuvenated T cells from tumor- or virus-specific targets *were generated* (Maeda et al., 2016; Saito et al., 2016; Vizcardo et al., 2013), including HIV (Nishimura et al., 2013).

Figure 3. Generation of macrophages from CCR5mut iPSCs

(A) Schematic representation of hematopoietic differentiation of iPSCs to MHPs and their further differentiation into macrophages.

(B) Day10 MHPs were cultured for 5 to 6 days in the presence of M-CSF and IL-1β to generate macrophages. The phenotype and morphology of the cells was confirmed by flow cytometry and Wright stain. Representative graphs and images of three independent experiments are shown. Scale bar, 50 μm.

(C and D) Genomic PCR to confirm *CCR5* mutation in Fib-iPSCs (C) and T-iPSCs (D). Biallelic *CCR5* mutation was maintained in the iPSCs, MHPs, macrophages, and T cells following differentiation as checked by genomic PCR.

(E) Loss of CCR5 expression was confirmed in macrophages and T cells from both WT and mutant clones by RT-PCR. β-actin was used as an internal control.

See also Figure S3.

To advance this strategy in a preclinical NHP model, we successfully generated T-iPSCs from MCM peripheral blood T cells, and demonstrated that T-iPSC can be successfully re-differentiated into T cells. However, we found that $CD34^+$ MHP generated from T-iPSC produced T cells less efficiently and CCR5mut-T-iPSCs did not generate $CD4^+CD8^+$ T cells, despite no apparent loss in the efficiency of hematopoietic differentiation and macrophage production from WT and CCR5mut-T-iPSCs. This defect in hematopoietic differentiation affected the T cell differentiation step only from T-iPSCs, but not fib-iPSCs, which reproducibly produced $CD4^+CD8^+$ T cells regardless of the presence or absence of the *CCR5* mutation. The reason for these differences remains unclear. It has been shown that following differentiation, T-iPSCs express TCR complex prematurely before the $CD4^+CD8^+$ double-positive stage (Maeda et al., 2016; Nagano et al., 2020), which may lead to strong TCR signaling and eventually death of T cell progenitors. In addition, previous studies have revealed that CCR5 expression promotes IL-2-dependent events during T cell activation, including IL-2R expression, STAT5 phosphorylation, and T cell proliferation (Camargo et al., 2009). Thus, it is possible that CCR5 deficiency in an environment of premature TCR expression may further contribute to the demise of T cell differentiation potential. Further focused studies are required to determine the exact mechanism(s) responsible for selective loss of $CD4^+CD8^+$ differentiation potential of T-iPSCs with edited CCR5.

In summary, we have shown that genomic editing of *CCR5* can be easily and effectively attained in NHP iPSCs that can be clonally selected to ensure homogeneous CRISPR-Cas9 gene editing. These lines can be useful for understanding the role of CCR5 in HIV pathogenesis and further advancement of iPSC-based technologies for an HIV cure in NHP preclinical models. We also noted that introduction of *CCR5* mutation into T-iPSCs affected their T cell redifferentiation potential. This unexpected finding presents an additional challenge to applying T cell rejuvenation technologies for HIV therapies using *CCR5*-edited HIV-resistant T-iPSCs.

EXPERIMENTAL PROCEDURES

NHP iPSC culture and generation of CCR5mut iPSC lines

All animal procedures were approved by the University of Wisconsin Medical School's Animal Care and Use Committee. MCM iPSCs were harvested using Collagenase IV followed by TrypLE to make a single cell suspension; 1×10^5 singularized cells were resuspended in 100 μL of nucleofector solution (Lonza) containing 10 μg of each sgRNA (#1 and #2 modified sgRNAs, Synthego) and 15 μg Cas9 protein (PNA Bio) and were electroporated using program A23 on the Nucleofector 2b Device (Lonza). After transfection, cells were replated onto MEFs (mouse embryonic fibroblasts) in Primate Embryonic Stem cell medium (ReproCELL) supplemented with 4 ng/mL bFGF (basic fibroblast growth factor) (154 amino acids.) (Peprotech). 15–20 days later, colonies were picked and expanded. Single cell derived knockout cell lines were obtained by single colony picking method with low-density iPSCs culture on MEFs. Genomic DNA from iPSC colonies was extracted using the Quick-DNA Miniprep kit (Zymo Research) and analyzed by PCR. The targeting genomic PCR in *CCR5*-mutated clones was performed using Q5 Hot Start High Fidelity DNA polymerase (NEB) with the following primers: P1 (TCAATGTGAAACAAATCGCAGC) and P2 (TCGTTTCGACACCGAAGCAG) CCR5-specific primers. Primers specific to β-actin (forward: 5′-GCAGGAGATGGCCACGGCGCC-3′, reverse: 5′-TCT CCTTCTGCATCCTGTCGGC-3′) were used for internal controls.

Differentiation to T lymphoid cells and macrophages

Hematopoietic differentiation of NHP iPSCs was performed on OP9 in the presence of CHIR99021, as previously described (D'Souza et al., 2016). Briefly, small cell aggregates of iPSCs were added to a prolonged culture of OP9 feeder in medium supplemented with 10% HyClone FBS (Cytiva) and 50 μM β-mercaptoethanol (MilliporeSigma); 4 μM of CHIR99021 (Peprotech) and 50 ng/mL VEGF (Peprotech) were added on day 1, for 2 days. The medium was changed and fresh medium was supplemented with 50 ng/mL VEGF. On day 6, an additional 5 mL of medium along with a hematopoietic cytokine cocktail consisting of 50 ng/mL SCF (Peprotech), 50 ng/mL of VEGF (Peprotech), 20 ng/mL of TPO (Thrombopoietin) (Peprotech), 20 ng/mL of IL-3 (Peprotech), and 20 ng/mL of IL-6 (Peprotech) was added to the co-culture. The co-culture was incubated for 10 days in standard conditions of 37°C and 5% CO_2. The phenotype of the cells was confirmed by flow cytometry using antibodies against CD45 (Miltenyi) and CD34 (BD Biosciences).

For lymphoid differentiation, the floating $CD45^+CD34^+$ MHPs were collected from day 10 of NHP iPSC/OP9 co-cultures, strained through a 70 μm cell strainer (ThermoFisher Scientific) and resuspended in a T cell differentiation medium consisting of αMEM (Gibco) supplemented with 20% HyClone FBS, 5 ng/mL IL-7 (Peprotech), 5 ng/mL Flt3-Ligand (Peprotech), and 10 ng/mL SCF (Peprotech). The cells were cultured on OP9-DLL4 for 2 weeks with weekly passage. The floating cells from T cell cultures were analyzed by flow cytometry using antibodies against CD3, CD4, CD7 (BD Biosciences), CD5, CD8, and TCRαβ (Biolegend) and used for subsequent SIV challenge. For intracellular staining, cells were fixed and permeabilized by resuspending the cell pellet in BD cytofix/cytoperm buffer (BD Biosciences) for 30 min on ice. The cells were then washed with 1xPerm Buffer (BD Biosciences) and stained with CD3 antibody for 30 min in the dark, washed, and analyzed using the MACSQuant Analyzer (Miltenyi Biotec) and FlowJo software (Tree star). Control staining with the appropriate isotype matched mouse monoclonal antibody and unstained controls were included to establish a threshold for positive staining.

For macrophage differentiation, day 10 MHPs from OP9/iPSC co-culture were suspended in IMDM (Gibco) with 10% HyClone FBS supplemented with 20 ng/mL of M-CSF (Peprotech) and 10 ng/mL of IL-1β for 5 to 7 days. Cell phenotype was confirmed by

Wright-Giemsa staining and flow cytometry using antibodies against CD45 (Miltenyi), CD14 (BD Biosciences), and HLA-DR (BD Biosciences). Cells from day 6 of macrophage culture were used for SIV challenge. See Table S1 for the complete list and description of antibodies used in this study.

SIV challenge of iPSC-derived T cells and macrophages

To determine whether the CCR5mut immune cells were resistant to infection, we challenged T cells and macrophages derived from WT fib-iPSC-M3/M3 and CCR5mutC6-fib-iPSC-M3/M3 with the CCR5-tropic SIV isolates SIVmac239 and SIVmac316 open SpX, previously shown to be T cell or macrophage-tropic, respectfully (Mori et al., 2000). The SIV stocks were purified by overlaying 127 ng (SIVmac316 open SpX) or 87 ng (SIVmac239) of Gag p27 on 100 μL of a 20% sucrose cushion and centrifuging at 21,000 × *g* for 1 h at 4°C. Media and sucrose were removed, and the virus pellet was resuspended in 70 μL of PBS. Cell infection was performed using magnetofection. Briefly, 30 μL of ViroMag R/L beads (OZ Biosciences) were added and incubated at room temperature for 15 min (Sacha and Watkins, 2010). During incubation 3×10^5 WT or CCR5-mut cells were placed in a single well of a 24-well plate and centrifuged at 530 × *g* for 5 min. The virus/bead mixture was then added dropwise to the cells and placed on a magnet for 1 h at 37°C. When the incubation was finished, the cells were pelleted and washed five times with 1 mL PBS. T cells from iPSCs were treated with 0.05% or 0.25% trypsin for 2 min at 37°C to remove bound but noninternalized virions. Then 1×10^5 of each virus/cell combination was placed into three wells of a 48-well plate containing growth media and incubated for 7 days at 37°C, 5% CO_2. Culture supernatants were sampled at days 1, 4, and 7 post-magnetofection. A p27 ELISA (Zeptometrix) was performed on each time point according to the manufacturer's instructions to determine the amount of virus produced in each well. See supplemental materials for additional details of the experimental procedure.

SUPPLEMENTAL INFORMATION

Supplemental information can be found online at https://doi.org/10.1016/j.stemcr.2022.03.003.

AUTHOR CONTRIBUTIONS

S.S.D. generated and characterized CCR5mut T-iPSCs and fib-iPSCs, analyzed iPSC hematopoietic differentiation potential, produced macrophages, interpreted experimental data, made figures and wrote the manuscript. A.K. generated iPSCs from T cells, characterized CCR5mut T-iPSCs, and generated T cells from iPSCs. J.W. performed SIV infection studies. M.A.P. and L.T. generated and characterized CCR5mut T-iPSCs. J.M. generated iPSCs from fibroblasts. H.J.K. designed CCR5 targeting gRNAs. S.T.D. assisted in generation and characterization of hematopoietic differentiation of iPSCs from M6/M6 animals. T.G. and J.A.T. advised on iPSC generation and characterization. M.R. and I.S. developed the concept, led and supervised studies, analyzed and interpreted data, and wrote the manuscript.

CONFLICTS OF INTERESTS

The authors declare no competing interests.

ACKNOWLEDGMENTS

This work is supported by funds from the National Institutes of Health, United States (R24OD021322, R01HL132891 and P51OD011106). The following reagent was obtained through the NIH AIDS Reagent Program, Division of AIDS, NIAID, NIH: SIV_{mac}316 open SpX from Dr. Ronald C. Desrosiers.

Received: June 2, 2021
Revised: March 2, 2022
Accepted: March 3, 2022
Published: March 31, 2022

REFERENCES

Ando, M., and Nakauchi, H. (2017). 'Off-the-shelf' immunotherapy with iPSC-derived rejuvenated cytotoxic T lymphocytes. Exp. Hematol. *47*, 2–12. https://doi.org/10.1016/j.exphem.2016.10.009.

Budde, M.L., Wiseman, R.W., Karl, J.A., Hanczaruk, B., Simen, B.B., and O'Connor, D.H. (2010). Characterization of Mauritian cynomolgus macaque major histocompatibility complex class I haplotypes by high-resolution pyrosequencing. Immunogenetics *62*, 773–780. https://doi.org/10.1007/s00251-010-0481-9.

Camargo, J.F., Quinones, M.P., Mummidi, S., Srinivas, S., Gaitan, A.A., Begum, K., Jimenez, F., VanCompernolle, S., Unutmaz, D., Ahuja, S.S., and Ahuja, S.K. (2009). CCR5 expression levels influence NFAT translocation, IL-2 production, and subsequent signaling events during T lymphocyte activation. J. Immunol. *182*, 171–182. https://doi.org/10.4049/jimmunol.182.1.171.

Chen, B., Gilbert, L.A., Cimini, B.A., Schnitzbauer, J., Zhang, W., Li, G.W., Park, J., Blackburn, E.H., Weissman, J.S., Qi, L.S., and Huang, B. (2013). Dynamic imaging of genomic loci in living human cells by an optimized CRISPR/Cas system. Cell *155*, 1479–1491. https://doi.org/10.1016/j.cell.2013.12.001.

Chen, Z., Kwon, D., Jin, Z., Monard, S., Telfer, P., Jones, M.S., Lu, C.Y., Aguilar, R.F., Ho, D.D., and Marx, P.A. (1998). Natural infection of a homozygous delta24 CCR5 red-capped mangabey with an R2b-tropic simian immunodeficiency virus. J. Exp. Med. *188*, 2057–2065.

D'Souza, S.S., Maufort, J., Kumar, A., Zhang, J., Smuga-Otto, K., Thomson, J.A., and Slukvin, , II.. (2016). GSK3beta inhibition promotes efficient myeloid and lymphoid hematopoiesis from non-human primate-induced pluripotent stem cells. Stem Cell Rep. *6*, 243–256. https://doi.org/10.1016/j.stemcr.2015.12.010.

Fu, Y., Sander, J.D., Reyon, D., Cascio, V.M., and Joung, J.K. (2014). Improving CRISPR-Cas nuclease specificity using truncated guide RNAs. Nat. Biotechnol. *32*, 279–284. https://doi.org/10.1038/nbt.2808.

Gautam, R., Carter, A.C., Katz, N., Butler, I.F., Barnes, M., Hasegawa, A., Ratterree, M., Silvestri, G., Marx, P.A., Hirsch, V.M., et al. (2007). In vitro characterization of primary SIVsmm isolates belonging to different lineages. In vitro growth on rhesus macaque cells is not predictive for in vivo replication in rhesus macaques. Virology *362*, 257–270. https://doi.org/10.1016/j.virol.2006.12.037.

Greene, J.M., Lhost, J.J., Hines, P.J., Scarlotta, M., Harris, M., Burwitz, B.J., Budde, M.L., Dudley, D.M., Pham, N., Cain, B., et al.

(2013). Adoptive transfer of lymphocytes isolated from simian immunodeficiency virus SIVmac239Deltanef-vaccinated macaques does not affect acute-phase viral loads but may reduce chronic-phase viral loads in major histocompatibility complex-matched recipients. J. Virol. *87*, 7382–7392. https://doi.org/10.1128/JVI.00348-13.

Hale, M., Mesojednik, T., Romano Ibarra, G.S., Sahni, J., Bernard, A., Sommer, K., Scharenberg, A.M., Rawlings, D.J., and Wagner, T.A. (2017). Engineering HIV-resistant, anti-HIV chimeric antigen receptor T cells. Mol. Ther. *25*, 570–579. https://doi.org/10.1016/j.ymthe.2016.12.023.

Mori, K., Rosenzweig, M., and Desrosiers, R.C. (2000). Mechanisms for adaptation of simian immunodeficiency virus to replication in alveolar macrophages. J Virol *74*, 10852–10859. https://doi.org/10.1128/jvi.74.22.10852-10859.2000.

Iriguchi, S., Yasui, Y., Kawai, Y., Arima, S., Kunitomo, M., Sato, T., Ueda, T., Minagawa, A., Mishima, Y., Yanagawa, N., et al. (2021). A clinically applicable and scalable method to regenerate T-cells from iPSCs for off-the-shelf T-cell immunotherapy. Nat. Commun. *12*, 430. https://doi.org/10.1038/s41467-020-20658-3.

Iwamoto, Y., Seki, Y., Taya, K., Tanaka, M., Iriguchi, S., Miyake, Y., Nakayama, E.E., Miura, T., Shioda, T., Akari, H., et al. (2021). Generation of macrophages with altered viral sensitivity from genome-edited rhesus macaque iPSCs to model human disease. Mol. Ther. Methods Clin. Dev. *21*, 262–273. https://doi.org/10.1016/j.omtm.2021.03.008.

Kang, H., Minder, P., Park, M.A., Mesquitta, W.T., Torbett, B.E., and Slukvin, , II.. (2015). CCR5 disruption in induced pluripotent stem cells using CRISPR/Cas9 provides selective resistance of immune cells to CCR5-tropic HIV-1 virus. Mol. Ther. Nucleic Acids *4*, e268. https://doi.org/10.1038/mtna.2015.42.

Karagiannis, P., Iriguchi, S., and Kaneko, S. (2016). Reprogramming away from the exhausted T cell state. Semin. Immunol. *28*, 35–44. https://doi.org/10.1016/j.smim.2015.10.007.

Kumar, A., Lee, J.H., Suknuntha, K., D'Souza, S.S., Thakur, A.S., and Slukvin, , II.. (2019). NOTCH activation at the hematovascular mesoderm stage facilitates efficient generation of T cells with high proliferation potential from human pluripotent stem cells. J. Immunol. *202*, 770–776. https://doi.org/10.4049/jimmunol.1801027.

Lam, S., and Bollard, C. (2013). T-cell therapies for HIV. Immunotherapy *5*, 407–414. https://doi.org/10.2217/imt.13.23.

Liu, R., Paxton, W.A., Choe, S., Ceradini, D., Martin, S.R., Horuk, R., MacDonald, M.E., Stuhlmann, H., Koup, R.A., and Landau, N.R. (1996). Homozygous defect in HIV-1 coreceptor accounts for resistance of some multiply-exposed individuals to HIV-1 infection. Cell *86*, 367–377. https://doi.org/10.1016/s0092-8674(00)80110-5.

Maeda, T., Nagano, S., Ichise, H., Kataoka, K., Yamada, D., Ogawa, S., Koseki, H., Kitawaki, T., Kadowaki, N., Takaori-Kondo, A., et al. (2016). Regeneration of CD8alphabeta T cells from T-cell-derived iPSC imparts potent tumor antigen-specific cytotoxicity. Cancer Res. *76*, 6839–6850. https://doi.org/10.1158/0008-5472.CAN-16-1149.

Mandal, P.K., Ferreira, L.M., Collins, R., Meissner, T.B., Boutwell, C.L., Friesen, M., Vrbanac, V., Garrison, B.S., Stortchevoi, A., Bryder, D., et al. (2014). Efficient ablation of genes in human hematopoietic stem and effector cells using CRISPR/Cas9. Cell Stem Cell *15*, 643–652. https://doi.org/10.1016/j.stem.2014.10.004.

Mock, U., Machowicz, R., Hauber, I., Horn, S., Abramowski, P., Berdien, B., Hauber, J., and Fehse, B. (2015). mRNA transfection of a novel TAL effector nuclease (TALEN) facilitates efficient knockout of HIV co-receptor CCR5. Nucleic Acids Res. *43*, 5560–5571. https://doi.org/10.1093/nar/gkv469.

Mussolino, C., Alzubi, J., Fine, E.J., Morbitzer, R., Cradick, T.J., Lahaye, T., Bao, G., and Cathomen, T. (2014). TALENs facilitate targeted genome editing in human cells with high specificity and low cytotoxicity. Nucleic Acids Res. *42*, 6762–6773. https://doi.org/10.1093/nar/gku305.

Nagano, S., Maeda, T., Ichise, H., Kashima, S., Ohtaka, M., Nakanishi, M., Kitawaki, T., Kadowaki, N., Takaori-Kondo, A., Masuda, K., and Kawamoto, H. (2020). High frequency production of T cell-derived iPSC clones capable of generating potent cytotoxic T cells. Mol. Ther. Methods Clin. Dev. *16*, 126–135. https://doi.org/10.1016/j.omtm.2019.12.006.

Naidu, Y.M., Kestler, H.W., 3rd, Li, Y., Butler, C.V., Silva, D.P., Schmidt, D.K., Troup, C.D., Sehgal, P.K., Sonigo, P., Daniel, M.D., et al. (1988). Characterization of infectious molecular clones of simian immunodeficiency virus (SIVmac) and human immunodeficiency virus type 2: persistent infection of rhesus monkeys with molecularly cloned SIVmac. J. Virol. *62*, 4691–4696. https://doi.org/10.1128/JVI.62.12.4691-4696.1988.

Nishimura, T., Kaneko, S., Kawana-Tachikawa, A., Tajima, Y., Goto, H., Zhu, D., Nakayama-Hosoya, K., Iriguchi, S., Uemura, Y., Shimizu, T., et al. (2013). Generation of rejuvenated antigen-specific T cells by reprogramming to pluripotency and redifferentiation. Cell Stem Cell *12*, 114–126. https://doi.org/10.1016/j.stem.2012.11.002.

Patel, S., Chorvinsky, E., Albihani, S., Cruz, C.R., Jones, R.B., Shpall, E.J., Margolis, D.M., Ambinder, R.F., and Bollard, C.M. (2018). HIV-specific T cells generated from naive T cells suppress HIV in vitro and recognize wide epitope breadths. Mol. Ther. *26*, 1435–1446. https://doi.org/10.1016/j.ymthe.2018.04.009.

Pedroza-Martins, L., Gurney, K.B., Torbett, B.E., and Uittenbogaart, C.H. (1998). Differential tropism and replication kinetics of human immunodeficiency virus type 1 isolates in thymocytes: coreceptor expression allows viral entry, but productive infection of distinct subsets is determined at the postentry level. J. Virol. *72*, 9441–9452. https://doi.org/10.1128/JVI.72.12.9441-9452.1998.

Sacha, J.B., and Watkins, D.I. (2010). Synchronous infection of SIV and HIV in vitro for virology, immunology and vaccine-related studies. Nat. Protoc. *5*, 239–246. https://doi.org/10.1038/nprot.2009.227.

Saito, H., Okita, K., Chang, A.E., and Ito, F. (2016). Adoptive transfer of CD8+ T cells generated from induced pluripotent stem cells triggers regressions of large tumors along with immunological memory. Cancer Res. *76*, 3473–3483. https://doi.org/10.1158/0008-5472.CAN-15-1742.

Samson, M., Libert, F., Doranz, B.J., Rucker, J., Liesnard, C., Farber, C.M., Saragosti, S., Lapoumeroulie, C., Cognaux, J., Forceille, C.,

et al. (1996). Resistance to HIV-1 infection in caucasian individuals bearing mutant alleles of the CCR-5 chemokine receptor gene. Nature *382*, 722–725. https://doi.org/10.1038/382722a0.

Sung, J.A., Patel, S., Clohosey, M.L., Roesch, L., Tripic, T., Kuruc, J.D., Archin, N., Hanley, P.J., Cruz, C.R., Goonetilleke, N., et al. (2018). HIV-specific, ex vivo expanded T cell therapy: feasibility, safety, and efficacy in ART-suppressed HIV-infected individuals. Mol. Ther. *26*, 2496–2506. https://doi.org/10.1016/j.ymthe.2018.08.015.

Tebas, P., Stein, D., Tang, W.W., Frank, I., Wang, S.Q., Lee, G., Spratt, S.K., Surosky, R.T., Giedlin, M.A., Nichol, G., et al. (2014). Gene editing of CCR5 in autologous CD4 T cells of persons infected with HIV. N. Engl. J. Med. *370*, 901–910. https://doi.org/10.1056/NEJMoa1300662.

Trotman-Grant, A.C., Mohtashami, M., De Sousa Casal, J., Martinez, E.C., Lee, D., Teichman, S., Brauer, P.M., Han, J., Anderson, M.K., and Zuniga-Pflucker, J.C. (2021). DL4-mubeads induce T cell lineage differentiation from stem cells in a stromal cell-free system. Nat. Commun. *12*, 5023. https://doi.org/10.1038/s41467-021-25245-8.

Tycko, J., Myer, V.E., and Hsu, P.D. (2016). Methods for optimizing CRISPR-cas9 genome editing specificity. Mol. Cell *63*, 355–370. https://doi.org/10.1016/j.molcel.2016.07.004.

Vizcardo, R., Masuda, K., Yamada, D., Ikawa, T., Shimizu, K., Fujii, S., Koseki, H., and Kawamoto, H. (2013). Regeneration of human tumor antigen-specific T cells from iPSCs derived from mature CD8(+) T cells. Cell Stem Cell *12*, 31–36. https://doi.org/10.1016/j.stem.2012.12.006.

Wiseman, R.W., Karl, J.A., Bohn, P.S., Nimityongskul, F.A., Starrett, G.J., and O'Connor, D.H. (2013). Haplessly hoping: macaque major histocompatibility complex made easy. ILAR J. *54*, 196–210. https://doi.org/10.1093/ilar/ilt036.

Wiseman, R.W., Wojcechowskyj, J.A., Greene, J.M., Blasky, A.J., Gopon, T., Soma, T., Friedrich, T.C., O'Connor, S.L., and O'Connor, D.H. (2007). Simian immunodeficiency virus SIVmac239 infection of major histocompatibility complex-identical cynomolgus macaques from Mauritius. J. Virol. *81*, 349–361. https://doi.org/10.1128/JVI.01841-06.

Xiao, Q., Chen, S., Wang, Q., Liu, Z., Liu, S., Deng, H., Hou, W., Wu, D., Xiong, Y., Li, J., and Guo, D. (2019). CCR5 editing by Staphylococcus aureus Cas9 in human primary CD4(+) T cells and hematopoietic stem/progenitor cells promotes HIV-1 resistance and CD4(+) T cell enrichment in humanized mice. Retrovirology *16*, 15. https://doi.org/10.1186/s12977-019-0477-y.

Xu, L., Wang, J., Liu, Y., Xie, L., Su, B., Mou, D., Wang, L., Liu, T., Wang, X., Zhang, B., et al. (2019). CRISPR-edited stem cells in a patient with HIV and acute lymphocytic leukemia. N. Engl. J. Med. *381*, 1240–1247. https://doi.org/10.1056/NEJMoa1817426.

Zhang, J.P., Li, X.L., Neises, A., Chen, W., Hu, L.P., Ji, G.Z., Yu, J.Y., Xu, J., Yuan, W.P., Cheng, T., and Zhang, X.B. (2016). Different effects of sgRNA length on CRISPR-mediated gene knockout efficiency. Sci. Rep. *6*, 28566. https://doi.org/10.1038/srep28566.

Stem Cell Reports, Volume *17*

Supplemental Information

Generation of SIV-resistant T cells and macrophages from nonhuman primate induced pluripotent stem cells with edited CCR5 locus

Saritha S. D'Souza, Akhilesh Kumar, Jason Weinfurter, Mi Ae Park, John Maufort, Lihong Tao, HyunJun Kang, Samuel T. Dettle, Thaddeus Golos, James A. Thomson, Matthew R. Reynolds, and Igor Slukvin

Supplemental Materials for D'Souza et al.

Generation of SIV resistant T cells and Macrophages from Nonhuman Primate Induced Pluripotent Stem Cells with Edited CCR5 locus

Supplemental Experimental Procedures

Reprogramming NHP cells and demonstration of pluripotency

Fibroblasts were obtained from skin punch biopsies of MCM CY1 with M3/M3 MHC genotype and MCM CY3 with M6/M6 MHC genotype, while peripheral blood samples were obtained from CY1, CY2, and CY3 with corresponding M3/M3, M1/M3 and M6/M6 MHC genotypes. Fibroblasts were obtained from skin biopsy and reprogrammed using a combination of oriP/EBNA-1 episomal vectors expressing the 6 reprogramming factors OCT4, KLF4, SOX2, MYC, NANOG and LIN28 (OKSMNL) by electroporation as described previously (D'Souza et al., 2016; Yu et al., 2009) to generate fibroblast iPSCs (Fib-iPSCs). Peripheral blood T cells were reprogrammed using the CytoTune™-iPS 2.0 Sendai Reprogramming Kit (ThermoFisher Scientific) to generate T-iPSCs (Seki et al., 2010). Briefly, peripheral blood mononuclear cells were first separated by Ficoll centrifugation and then MACS enriched for $CD3^+$ cells. These cells were then activated using CD3 and CD28 antibodies in the presence of 100U/ml IL-2 (Peprotech). For reprogramming, $0.5x10^6$ activated cells were resuspended in 0.3ml of 10% RPMI1640 medium (ThermoFisher) containing 4μg/ml of hexadimethrine bromide (MilliporeSigma) and 20μl each of the 3 Sendai viruses expressing KOS (KLF4–OCT3/4–SOX2), MYC, and KLF4, and incubated overnight at 37^oC in 5% CO_2. Cells were washed the following day and transferred onto mouse embryonic fibroblasts (MEFs). Colonies began to appear within 15 days and were subsequently transferred onto fresh MEFs for expansion and characterization. The iPSC lines (T-iPSCs and Fib-iPSCs) were maintained on MEFs in Primate ES cell medium (ReproCELL) supplemented with 4ng/ml bFGF (154 a.a.) (Peprotech) as previously described.(D'Souza *et al.*, 2016) Cells were passaged every 3-4 days using Collagenase Type IV (Life Technologies). Expression of pluripotency markers was analyzed by flow cytometry using SOX2 (Cell Signaling) and OCT3/4 (Santacruz Biotechnology) antibodies and the MACSQuant Analyzer 10 (Miltenyi) and FlowJo software (BD). Expression of pluripotency marker NANOG was analyzed by immunofluorescence using antibodies from Cell Signaling.

RT-PCR analysis of CCR5 expression

RNA was isolated using the RNeasy mini Kit (Qiagen) from macrophages and T cells generated from wild type and CCR5-mut iPSCs. cDNA was transcribed using the QuantiTect reverse transcription kit (Qiagen) from all the samples and amplified by PCR using Q5 master mix (New England Biolabs) and forward-TGTGTCAATGGAACTCTTGAC and reverse-TCGTTTCGACACCGAAGCAG CCR5 primers.

Supplemental References

D'Souza, S.S., Maufort, J., Kumar, A., Zhang, J., Smuga-Otto, K., Thomson, J.A., and Slukvin, II (2016). GSK3beta Inhibition Promotes Efficient Myeloid and Lymphoid Hematopoiesis from Non-human Primate-Induced Pluripotent Stem Cells. Stem Cell Reports *6*, 243-256. 10.1016/j.stemcr.2015.12.010.

Seki, T., Yuasa, S., Oda, M., Egashira, T., Yae, K., Kusumoto, D., Nakata, H., Tohyama, S., Hashimoto, H., Kodaira, M., et al. (2010). Generation of induced pluripotent stem cells from human terminally differentiated circulating T cells. Cell Stem Cell *7*, 11-14. 10.1016/j.stem.2010.06.003.

Yu, J., Hu, K., Smuga-Otto, K., Tian, S., Stewart, R., Slukvin, II, and Thomson, J.A. (2009). Human induced pluripotent stem cells free of vector and transgene sequences. Science *324*, 797-801. 10.1126/science.1172482.

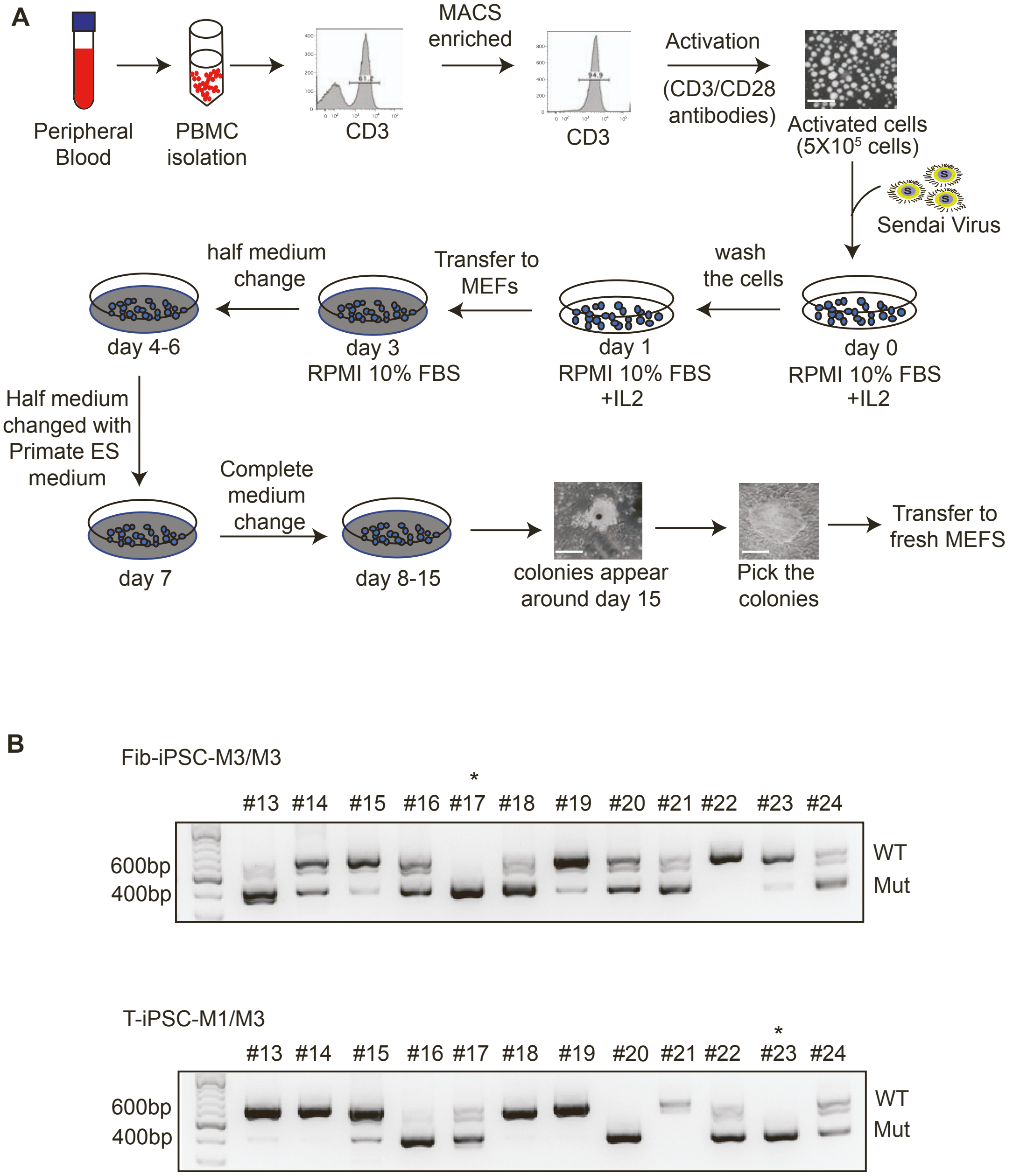

Supplementary Figure S1, related to Figure 1. Generation of CCR5mut iPSCs from MCM fibroblasts and T cells. (A) Schematic diagram of generation of T-iPSCs from peripheral blood T cells using Sendai virus kit. Scale bar in activated T cell images is 300 µm. Scale bar for colonies at day 15 is 150 µm and for pick up colonies is 740 µm. (B) Genomic PCR to detect deletion within CCR5 locus. * denotes clones selected in these studies.

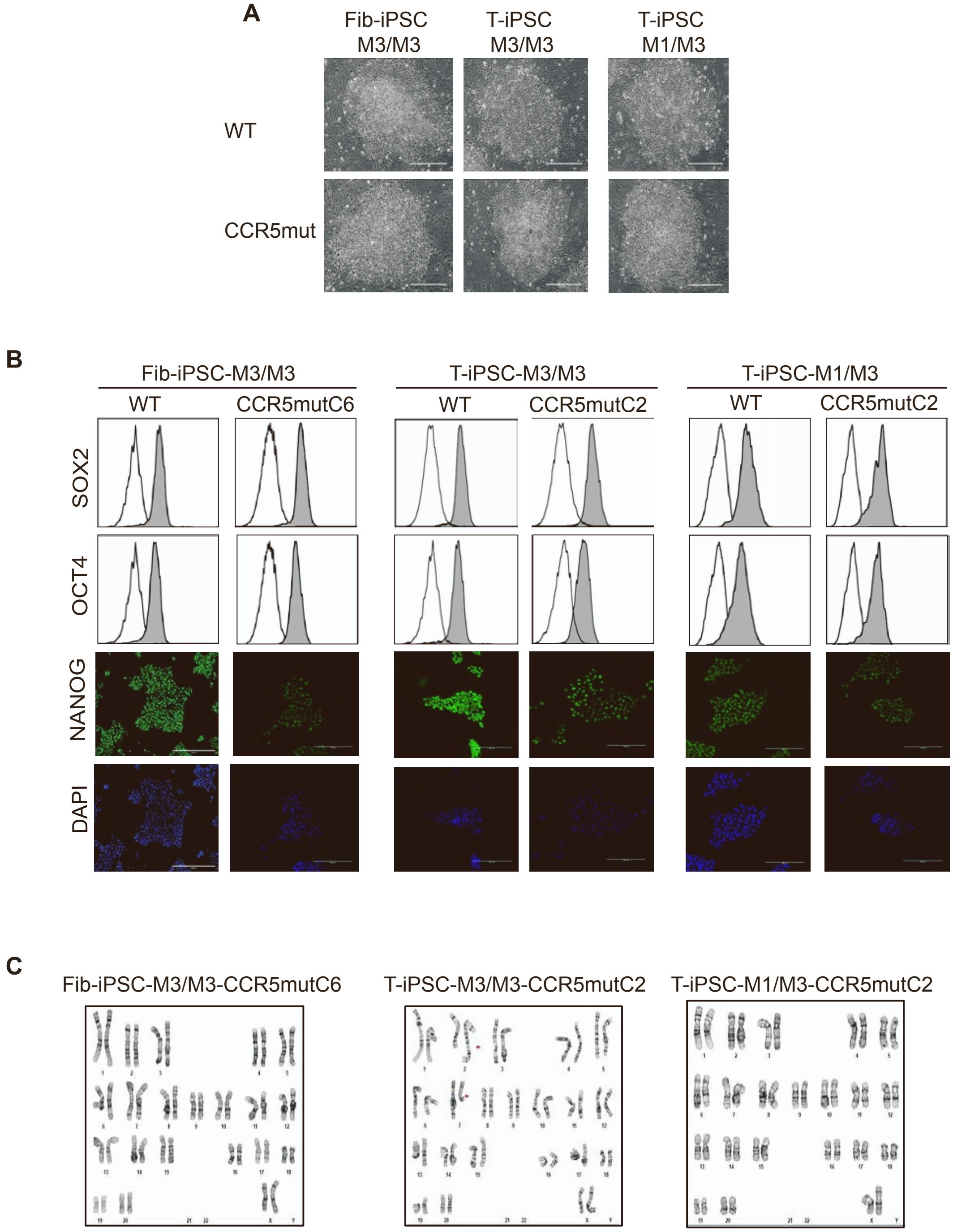

Supplementary Figure S2, related to Figure 1. Generation and characterization of MCM-iPSCs. (A) Phase contrast images of WT and CCR5mut colonies on MEFs. Scale bar is 200 µm. (B) Expression of pluripotency markers by flow cytometry (OCT4 and SOX2) and by immunofluorescence (NANOG) in generated wild type and CCR5mut iPSC lines. Scale bars for WT Fib-iPSC-M3/M3 images are 400 µm. Scale bars for all other images are 100 µm. (C) Karyotype of CCR5mut iPSCs.

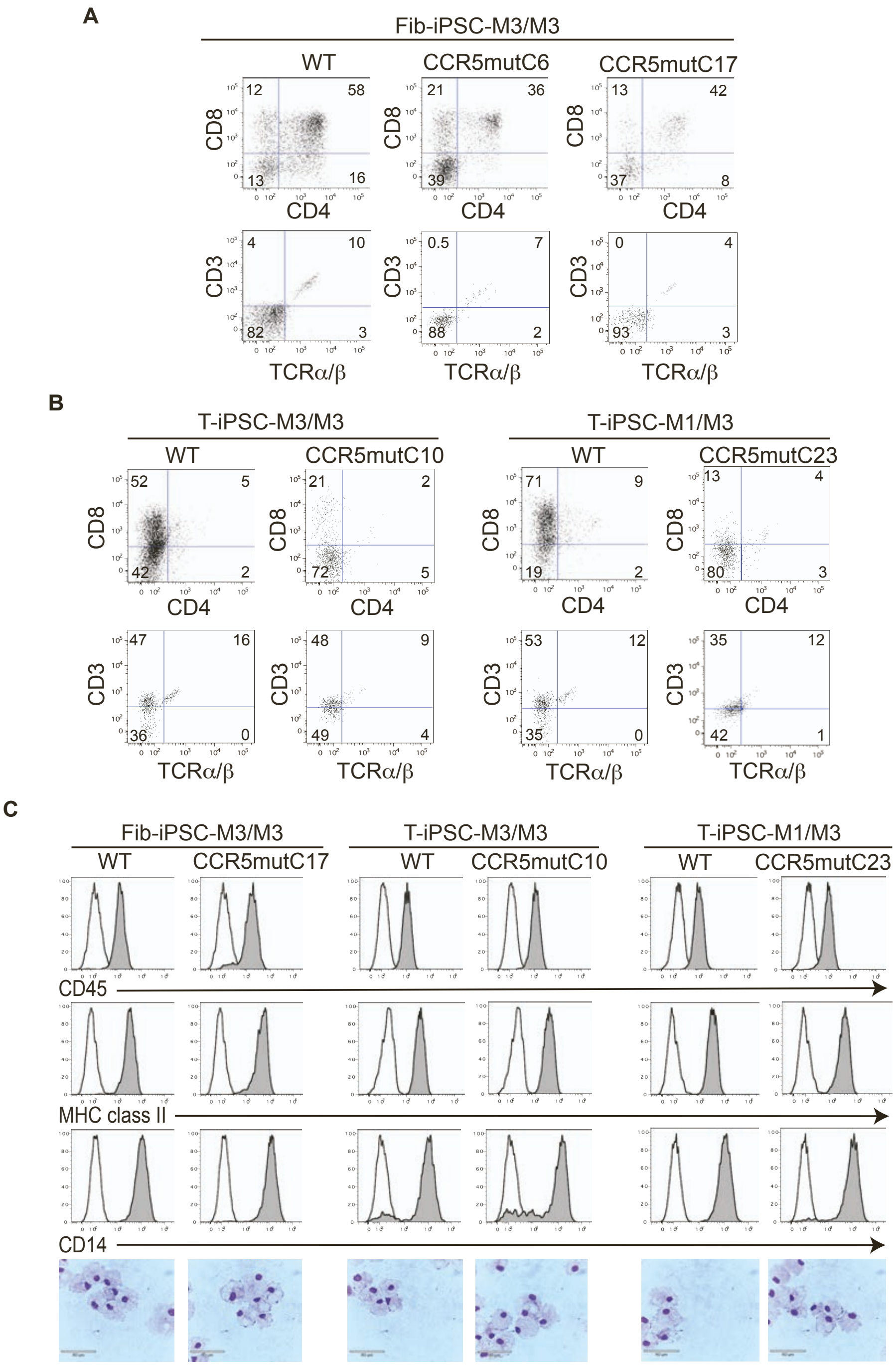

Supplemental Figure S3, related to Figure 2 and 3. Generation of T cells and macrophages from CCR5mut clones. (A) CCR5mutC6 and C17 clones from fib-iPSCs and (B) C10 and C23 CCR5mut clones from T-iPSC-M3/M3 and M1/M3 were differentiated into T cells. Expression of T cell markers was shown on $CD45^+$ gated cells after 3 weeks of culture of iPSC-derived MHP on OP9-DLL4 in the presence of SCF, IL7 and FLT3L. (C) Day 10 $CD34^+CD45^+$ MHPs from mutant and wild type NHP iPSCs were differentiated to macrophages. The phenotype and morphology of the cells was confirmed by flow cytometry and Wright stain of cytospins. Scale bar is 50 µm.

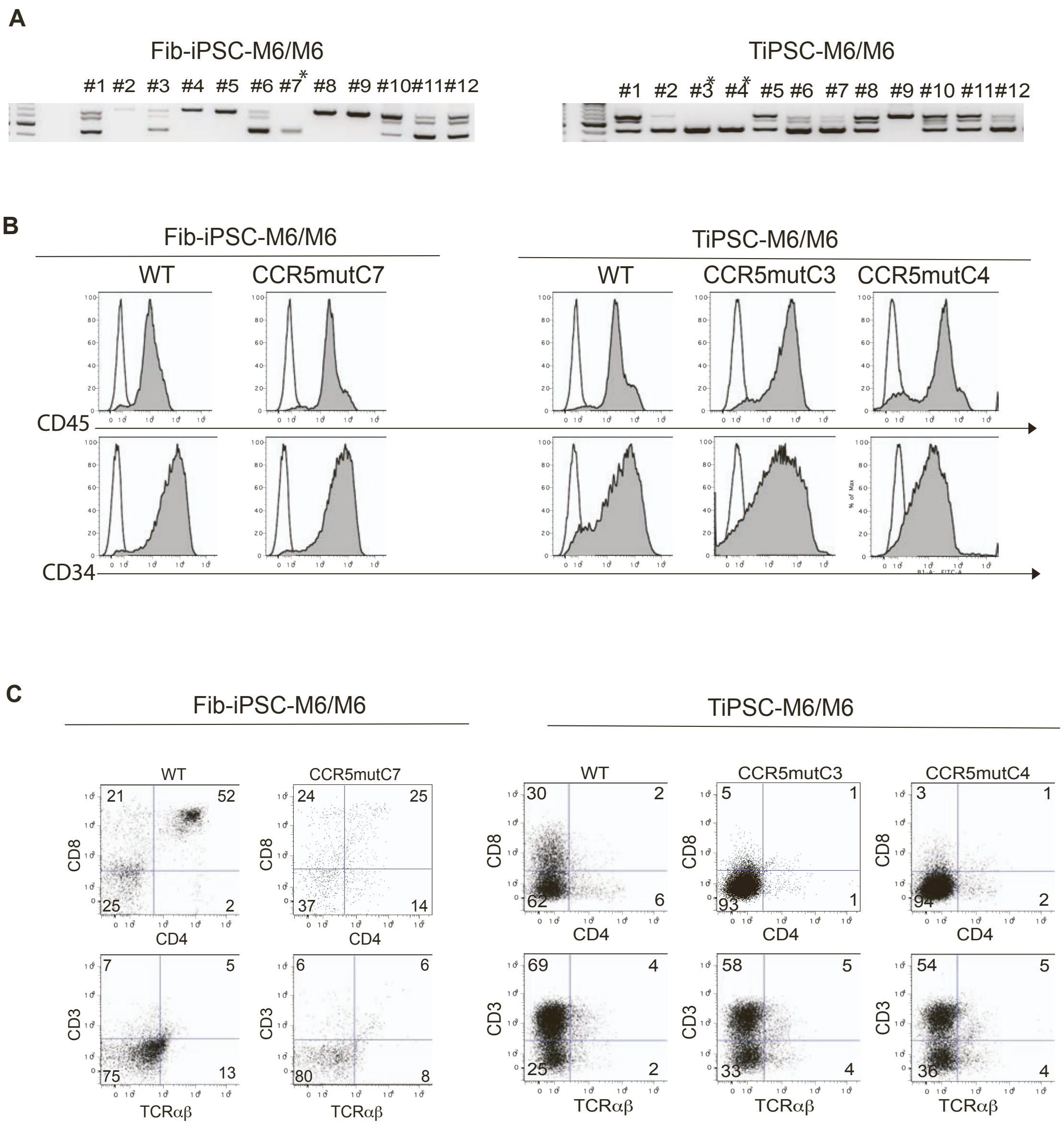

Supplemental Figure S4, related to Figure 1, 2 and 3. Generation of fib- and T-iPSCs from MCM with M6/M6 genotype and their hematopoietic differentiation. (A) Genomic DNA was isolated from single cell clones obtained after CCR5 editing and analyzed by PCR to detect deletion within the CCR5 locus. * Denotes the clones selected for further analysis. (B) Both wild type and CCR5mut, fib- and T-iPSCs efficiently generate $CD34^{+}CD45^{+}$ hematopoietic progenitors as analyzed by flow cytometry of floating cells collected on day10 of iPSC/OP9 cocultures. (D) $CD34^{+}CD45^{+}$ cells from WT and CCR5mut iPSCs were cultured on OP9-DLL4 in the presence of SCF, IL7 and FLT3L for 3 weeks and floating cells were analyzed by flow cytometry after gating on $CD45^{+}$ cells. Control staining with appropriate isotype matched antibodies were included to establish a threshold for positive staining. The graphs are representative of at least 3 independent experiments.

Supplementary Table S1. List of antibodies used in this study

Name	Clone	Vendor	Cat. Number
Anti NHP CD45	MB4-6D6	Miltenyi Biotech	130-119-764
Anti human CD34	563	BD Biosciences	550619
Anti human CD3ε	SP34	BD Biosciences	556612
Anti human CD4	L200	BD Biosciences	550630
Anti human CD5	UCHT2	Biolegend	300611
Anti human CD7	MT701	BD Biosciences	340656
Anti rat TCRα/β	R73	Biolegend	201110
Anti human CD8	SK1	Biolegend	980904
Anti human CD14	M5E2	BD Biosciences	555399
Anti human MHC class II	L243	BD Biosciences	340689
Anti monkey CD3-1	monoclonal	Mabtech	3610-1-50
Anti human CD28-A	monoclonal	Mabtech	3608-1-50
Oct 3/4	monoclonal	Santa Cruz	Sc-5279
Donkey anti mouse IgG (H+L) Alexa Fluor®488	polyclonal	Invitrogen	A-31570
Nanog	monoclonal	Cell Signaling Technology	4903
Donkey anti rabbit IgG (H+L) Alexa Fluor®488	polyclonal	Invitrogen	A-21206
Sox 2	monoclonal	Cell Signaling Technology	3579
Donkey anti rabbit IgG (H+L) Alexa Fluor®555	polyclonal	Invitrogen	A-31572
